# Investigation of risk factors for introduction of highly pathogenic avian influenza H5N1 infection among commercial turkey operations in the United States, 2022: a case-control study

**DOI:** 10.3389/fvets.2023.1229071

**Published:** 2023-08-30

**Authors:** Kelly A. Patyk, Victoria L. Fields, Andrea L. Beam, Matthew A. Branan, Rachel E. McGuigan, Alice Green, Mia K. Torchetti, Kristina Lantz, Alexis Freifeld, Katherine Marshall, Amy H. Delgado

**Affiliations:** ^1^Center for Epidemiology and Animal Health, Animal and Plant Health Inspection Service, United States Department of Agriculture, Fort Collins, CO, United States; ^2^National Veterinary Services Laboratories, Animal and Plant Health Inspection Service, United States Department of Agriculture, Ames, IA, United States

**Keywords:** avian influenza, biosecurity, case control, H5N1, highly pathogenic avian influenza, risk factors, turkey

## Abstract

**Introduction:**

The 2022–2023 highly pathogenic avian influenza (HPAI) H5N1 outbreak in the United States (U.S.) is the largest and most costly animal health event in U.S. history. Approximately 70% of commercial farms affected during this outbreak have been turkey farms.

**Methods:**

We conducted a case-control study to identify potential risk factors for introduction of HPAI virus onto commercial meat turkey operations. Data were collected from 66 case farms and 59 control farms in 12 states. Univariate and multivariable analyses were conducted to compare management and biosecurity factors on case and control farms.

**Results:**

Factors associated with increased risk of infection included being in an existing control zone, having both brooders and growers, having toms, seeing wild waterfowl or shorebirds in the closest field, and using rendering for dead bird disposal. Protective factors included having a restroom facility, including portable, available to crews that visit the farm and workers having access and using a shower at least some of the time when entering a specified barn.

**Discussion:**

Study results provide a better understanding of risk factors for HPAI infection and can be used to inform prevention and control measures for HPAI on U.S. turkey farms.

## 1. Introduction

Avian influenza viruses (AIV) are distributed worldwide (1, 2). Wild waterfowl are primary natural reservoirs and have an important role in the maintenance and dispersal of AIVs, including H5 and H7 subtypes, that have the potential to result in outbreaks in domestic poultry (3–5). Spillover of AIVs from wild birds to poultry may occur through direct (i.e., direct exposure to birds infected with AIV) or indirect (e.g., exposure to contaminated soil, water, fomites, aerosols, or droplets) routes of transmission (6, 7). Outbreaks of AIVs in domestic poultry can result in high morbidity and mortality among poultry and serious economic impacts due to the loss of birds from death or depopulation, outbreak response costs, and trade restrictions (8).

On 20 December 2021, HPAI H5N1 was detected in a mixed species flock on an exhibition farm in Newfoundland, Canada, following a period of rapid, increased mortality in the flock, and retrospective testing identified virus in a wild black-backed gull from a nearby pond that had died in November 2021 (9). Phylogenetic analysis indicated that these A/Goose/Guangdong/1/1996 lineage (GsGD) viruses belonged to HPAI clade 2.3.4.4b and were likely spread to Newfoundland from Europe by migratory birds (9). In late December 2020 and January 2021, GsGD lineage clade 2.3.4.4b H5N1 HPAI was detected in several wild bird species sampled in the Atlantic Flyway in North Carolina and South Carolina as part of the routine AIV surveillance program (10). The first U.S. commercial poultry flock was detected in Indiana in February of 2022, and detections of Eurasian H5 2.3.4.4b GsGD viruses have subsequently occurred in commercial and backyard poultry flocks, wild birds, and wild mammals across the United States (11, 12).

In 2022 alone, over 57 million commercial and backyard poultry on over 700 farms across 47 U.S. states were affected, resulting in over $659 million in federal expenditures for control efforts and indemnity payments. Commercial turkey farms comprised the highest percentage of affected commercial poultry farms, with approximately 70% of all affected commercial farms being turkey farms. Results of full genome sequencing indicated that independent wild bird introductions were the primary mechanism of introduction of virus into operations in this outbreak (Youk et al., in preparation). In comparison, the severity of the 2014–2015 U.S. HPAI H5N2 and H5N8 outbreak was heavily influenced by lateral transmission of virus between farms (13, 14). Several studies conducted during the 2014–2015 outbreak explored potential risk factors for transmission of virus within and between farms (15–17). The differences in spread mechanism, as well as the larger geographic scope of the 2022 outbreak, as compared to the 2014–2015 outbreak, necessitated further examination into risk factors for introduction and biosecurity practices.

The goal of this study was to investigate potential risk factors for introduction of HPAI virus onto commercial turkey farms. To address this goal, the United States Department of Agriculture (USDA) Animal and Plant Health Inspection Service (APHIS), with support from the USDA National Agricultural Statistics Service (NASS), as well as from State and national poultry organizations, conducted a case-control study among commercial meat turkey operations. The study objectives were to (1) identify risk factors for infection with HPAI, (2) identify biosecurity challenges on turkey farms, and (3) refine biosecurity recommendations to support prevention of infection on farms. This information will improve understanding of the risk factors associated with introduction of HPAI on turkey farms and will be valuable for informing enhancements of on-farm preventive measures.

## 2. Materials and methods

### 2.1. Study design

A case-control study was designed to examine risk factors associated with HPAI infection on U.S. commercial turkey farms. Commercial turkey farms that raised meat turkeys between 1 January and 17 October 2022, and that raised more than 30,000 meat turkeys annually, were eligible to participate in the study. Commercial turkey breeder farms and backyard farms with turkeys were excluded from the study.

Case farms were defined as commercial meat turkey farms that met the USDA’s HPAI case definition during the study time frame (18). Farms were tested for HPAI during the outbreak in accordance with USDA HPAI response plans. For farms being tested, samples were screened for influenza A and H5/H7 subtypes by real-time reverse transcription polymerase chain reaction (RT-PCR) by members of the National Animal Health Laboratory Network (NAHLN). Samples testing non-negative by influenza A virus (IAV) PCR were forwarded to the National Veterinary Services Laboratories (NVSL, Ames, Iowa, United States) for confirmation. Testing at NVSL included an H5 clade 2.3.4.4 pathotyping assay and an assay targeting N1 for neuraminidase subtyping and whole genome sequencing was conducted directly from the samples. Influenza A viruses were sequenced directly from samples as previously described (10), RAxML was used to generate phylogenetic trees, and tables of single nucleotide polymorphisms (SNPs) were created using the vSNP pipeline.^1^ For purposes of the case-control study, the reference date was the date of onset of clinical signs, or if not available/applicable, the date of a presumptive diagnosis based on the USDA’s case definition (18) on the farm.

Control farms were defined as commercial meat turkey farms that did not meet the USDA’s case definition for HPAI during the study period. For each case farm, 2 to 5 control farms located in the same state were randomly selected. Enumerators were instructed to move to the next case after they had gotten 1 to 2 completed controls for a single case. Contact information for case and control farms was obtained from the USDA Veterinary Services Emergency Management Response System (EMRS), from Thomson Reuters® CLEAR software, from State databases where available, and from poultry company representatives. At the start of the study, the potential sampling pool consisted of 161 HPAI-affected commercial meat turkey farms in 13 states (CA, IA, IN, KY, MI, MN, MO, NC, ND, PA, SD, UT, WI). A total of 153 case farms from all 13 states were contacted for participation. Eight case farms were excluded due to a lack of availability of contact information within the study timeline.

### 2.2. Data collection and sources

A 24-page questionnaire (Supplementary material) was administered to farm managers or supervisors on each participating farm by telephone interview by trained NASS enumerators or USDA–APHIS epidemiologists, or by mail. The questions focused on farm characteristics, wild birds and wildlife, biosecurity, personnel, visitors, vehicles and equipment, and management practices for the 14 days prior to the reference date on a case farm and a comparable 14-day reference period on control farms. The length of the reference period was chosen based on the flock-level 14-day incubation period recognized by the World Organization for Animal Health for HPAI (19). Control farms were provided with a tentative 14-day reference period that was the same as the 14-day reference period for a case farm located in the same state. If a control farm did not have turkeys on the farm for the tentative 14-day period, they were asked to identify the closest 14-day period to the tentative reference period in 2022 during which they had turkeys on the farm. The 14 days identified were then used as the 14-day reference period when answering questions. Some questions asked about practices for the entire farm, and some asked about practices for a “selected barn.” The selected barn on case farms was the first barn on the farm to be confirmed HPAI positive, and for control farms, respondents were asked to identify a single barn at random to be designated as the selected barn.

Data collection took place between 7 November 2022 and 27 February 2023. Following data entry, survey responses were validated to identify logical inconsistencies in the data. Validation identified numeric extremes, improper categorical responses, and erroneous skip patterns, and relational checks were performed. Errors were evaluated by two analysts. Where deductions from other survey responses could not be made, appropriate solutions were implemented as agreed upon by the two analysts, and, for some errors, enumerators followed up with producers or cross-checked reported results using EMRS.

The zone status for each case and control farm during their 14-day reference period was determined using information from EMRS. Following detection of infection of HPAI on a farm, a 10 km radius control zone is established around the infected farm for purposes of outbreak response. Zones remain in place for a duration consistent with the HPAI outbreak response plan, typically 4 to 5 weeks for this outbreak. Zone status (i.e., whether a farm was located inside or outside of an existing control zone) was included as a covariate in the multivariable analysis.

### 2.3. Statistical analyses

Data were analyzed to identify statistical associations between infected status (case vs. control) and farm or selected barn characteristics, such as management practices. The percentages of case and control farms having each characteristic were calculated. Univariate analyses were performed to identify variables potentially associated with the presence of HPAI at the farm/selected barn level.

For the univariate analyses, Fisher’s exact test was used for categorical variables and the Score test for continuous variables to assess the association of each variable with HPAI infection. Variables with *p*-values ≤ 0.20 and where the relationship was biologically plausible for risk of HPAI infection were considered for entry into candidate multivariable models.

The subsets of farms that had either lateral transmission/common source or wild bird introduction were evaluated via univariate analyses, while a multivariable model was only created for wild bird introduction due to the low number of cases associated with lateral transmission/common source exposure between farms.

To address item non-response, random, single imputation was performed on the variables that entered the multiple logistic regression model as candidate variables. Multivariable results are reported using the imputed data, whereas univariate results are reported using the non-imputed data. Hierarchical cluster analysis of predictor variables using PROC VARCLUS was used to help guide final model selection, and variance inflation factors (VIFs) were computed to help identify issues with multicollinearity, with VIFs exceeding 3 indicating further investigation was needed (20).

Multivariable logistic regression models were fit using PROC LOGISTIC in SAS version 9.4. Forward-, backward-, and step-wise selection procedures were carried out via PROC HPGENSELECT to select a final model from which to make inference, using the AICc criterion, which is a variant of Akaike’s information criterion with an adjustment for small sample sizes (21, 22). The final model results using imputation matched the results using the non-imputed data. Primary model outputs included estimated odds ratios (ORs) and their 95% confidence intervals, along with Type III F-test *p*-values to assess statistical significance of effects (23). Factors were considered statistically significant in final multivariable models if *p* < 0.05.

All statistical analyses were conducted using SAS version 9.4 (SAS Institute Inc., Cary, NC, United States).

## 3. Results

Questionnaires were completed for 67 case farms and 61 control farms across 12 states (CA, IA, IN, MI, MN, MO, NC, ND, PA, SD, UT, WI). One case and one control questionnaire were excluded because the farms had only breeder turkeys on-site during the 14-day reference period. One case farm completed both a case and a control questionnaire; the control questionnaire was excluded from this analysis. After excluding the farms without meat turkeys and adjusting for case-control status, there were completed questionnaires for 66 case farms and 59 control farms across 12 states. The sample included 30 company farms, 50 contract farms including lessees, and 44 independent farms; 1 farm had a missing response for this question.

Case farms had a median of 37,356 birds (range: 8,000–300,000), and control farms had a median of 39,350 birds (range: 6,000–200,000). The phylogenetic analyses provided evidence for independent introductions of virus from wild birds on 77% of case farms (*n* = 51) and suggested lateral spread or common source exposure on 23% of case farms (*n* = 15).

### 3.1. Univariable analysis

Selected results from the univariate analyses are shown in Tables 1–5. A complete list of univariable results is available in Supplementary material.

**Table 1:** Univariate analyses of premises characteristics (*p* ≤ 0.20) considered for entry into the multivariable model.

Characteristic	Level	Number of case farms (%)	Number of control farms (%)	Univariate *p*-value
In an existing control zone^A^		21 (31.8)	7 (11.9)	0.01
Stage of production	Only brooder or only grower	32 (48.5)	43 (72.9)	<0.01
	Brooder and grower	34 (51.5)	16 (27.1)	
Hens on the farm		14 (21.2)	28 (48.3)	<0.01
Toms on the farm		57 (86.4)	39 (67.2)	0.02
Other poultry on farm		13 (19.7)	3 (5.2)	0.02
Farm type	Independent	29 (43.9)	15 (25.9)	0.04
	Other	37 (56.1)	43 (74.1)	
Wetland or swamp within 320 m (350 yards) of farm		17 (25.8)	8 (13.8)	0.12

Percentage of case farms and percentage of control farms by premises characteristics. ^A^During the 14-day reference period.

Percentage of case farms and percentage of control farms by premises characteristics.

**Table 2:** Univariate analyses of wild animal characteristics (*P* ≤ 0.20) considered for entry into the multivariable model.

Characteristic^A^	Number of case farms (%)	Number of control farms(%)	Univariate *p*-value
Waterfowl/shorebirds seen on bodies of water within 320 m (350 yards) of farm^B^	23 (34.8)	9 (15.3)	0.01
Waterfowl/shorebirds seen on closest body of water^B^	30 (45.5)	14 (23.7)	0.01
Waterfowl/shorebirds seen in closest field^B^	20 (30.3)	7 (11.9)	0.02
Waterfowl seen within 91.4 m (100 yards) of the barns^C^	33 (51.6)	16 (27.6)	<0.01
Wild mammals near barns^D^	13 (21.3)	19 (35.2)	0.14

Percentage of case farms and percentage of control farms by wild animal characteristics. ^A^During the 14-day reference period.

B Any birds present (tens, hundreds, thousands) vs. none/do not know.

C Birds often or sometimes seen within 91.4 m (100 yards) of the barn vs. never.

D Yes vs. no.

**Table 3:** Univariate analyses of biosecurity practices (*p* ≤ 0.20) considered for entry into the multivariable model.

Biosecurity practice	Level	Number of case farms (%)	Number of control farms (%)	Univariate *p*-value
Workers showered^A^		7 (10.8)	15 (25.9)	0.04
Workers washed hands or used hand sanitizer^A^		54 (83.1)	53 (91.4)	0.19
Visitor log usedRestroom facility available to crews visiting farm	Always	39 (60.0)	45 (77.6)	0.05
	Sometimes/never	26 (40.0)	13 (22.4)	
	Always/sometimes	30 (45.5)	41 (69.5)	0.02
	Never	26 (39.4)	12 (20.3)	
	Unknown (skipped question)	10 (15.2)	6 (10.2)	

Percentage of case farms and percentage of control farms by biosecurity practices.

^A^Workers always, most of the time, or sometimes used the practice before entering the barn vs. never or not available.

This question was asked specifically for the selected barn and for the 14-day reference period.

**Table 4:** Univariate analyses of management practices and vehicle characteristics (*p* ≤ 0.20) considered for entry into the multivariable model.

Characteristic	Level	Number of case farms (% ) or median^*^	Number of control farms (%) or median^*^	Univariate *p*-value
Bird drinking water treated (e.g., chlorination)	Continuously treated	60 (90.9)	46 (80.7)	0.10
	Intermittently/not treated	6 (9.1)	11 (19.3)	
Number vehicles entering the farm per week		5.0^*^	4.0^*^	0.10
Render dead birds^A^		18 (28.1)	8 (13.8)	0.08
Incinerate dead birds^A^		4 (6.3)	10 (17.2)	0.09
Landfill for dead bird disposal^A^		**	4 (6.9)	0.19
Vehicles for rendering	Come to the farm^B^	20 (32.3)	11 (19.0)	0.14

Percentage of case farms and percentage of control farms by management practices and vehicle characteristics. ^A^During the 14-day reference period. ^B^Includes to the perimeter of the farm, enter the farm but not near the barns, and come near the barns.

* Median.

** Too few to report.

**Table 5:** Univariate analyses of selected factors of interest not found to be associated with HPAI H5N1 infection (*p* > 0.20).

Characteristic	Level	Number of case farms (%)	Number of control farms (%)	Univariate *p*-value
Non-asphalt roads		62 (96.9)	56 (96.6)	1.00
Use of vehicle wash/spray station^A^		40 (60.6)	40 (69.0)	0.35
Closest field actively worked (e.g., tilled)^A^		6 (9.8)	6 (13.3)	0.76
Wild birds observed around dead bird collection area^A^		20 (32.8)	18 (31.6)	1.00
Ventilation type^B^	Curtain ventilated	29 (46.0)	22 (40.7)	0.90
	Environmental control/tunnel ventilation	16 (25.4)	14 (25.9)	
	Side doors	3 (4.8)	4 (7.4)	
	Other	15 (23.8)	14 (25.9)	
Percentage of time curtains open (for curtain ventilated)^A^^,^^B^	Less than 20%	7 (25.0)	3 (13.6)	0.48
	20% or more	21 (75.0)	19 (86.4)	
Use of landscape fabric on air inlets or along curtains^A^^,^^B^	Not used	44 (71.0)	36 (66.7)	0.72
	Used without disinfectant spray	8 (12.9)	10 (18.5)	
	Used with disinfectant	10 (16.1)	8 (14.8)	

A During the 14-day reference period.

B This question was asked specifically for the selected barn.

#### 3.1.1. Premises characteristics

During the 14-day reference period, more case farms were located within an existing control zone compared to control farms (32% vs. 12%, *p =* 0.01; Table 1). Having both brooder and grower production on the farm was associated with case status (52% vs. 27%; *p* < 0.01). Also, having toms as the market-type bird on the farm was associated with case status (86% vs. 67%; *p* = 0.02).

#### 3.1.2. Wild animal characteristics

In the univariate analysis, several variables were related to wild birds and nearby water bodies (Table 2). Cluster analysis showed several of these variables clustered together and were candidates for the multivariable analysis.

Wild waterfowl or shorebirds were seen in the closest field during the 14-day reference period on 30% of case farms, compared to 12% of control farms (*p* = 0.02). During the 14-day reference period, case farms also reported more commonly seeing wild waterfowl or shorebirds on water bodies found within 320 meters (350 yards) of the farm (35% vs. 15%; *p* = 0.01), seeing wild waterfowl or shorebirds on the closest body of water (45% vs. 24%; *p* = 0.01), and seeing waterfowl within 91.4 meters (100 yards) of the outside of the barns (52% vs. 28%; *p* < 0.01). Case farms also more commonly reported a wetland or swamp visible or within 320 meters (350 yards) of the farm (Table 1; 26% vs. 14%; *p* = 0.12). Control farms were more likely to see wild mammals (such as raccoons, opossums, skunks, coyotes, or foxes) or evidence of them in or around the barns (35% vs. 21%, *p* = 0.14).

#### 3.1.3. Biosecurity characteristics

Having a restroom facility (including portable) always or sometimes available to crews that visit the farm was more common on control farms compared to case farms (70% vs. 46%; *p* = 0.02; 13% of respondents did not answer this question; Table 3). Control farms were more likely to always use a visitor log (78% vs. 60%; *p* = 0.05).

Neither case nor control farms had many visitors overall, and there were no notable significant differences in the types of visitors coming to the farm. If visitors came onto the farm, they also had limited access to the turkey barns. There were not many differences reported in worker biosecurity practices. Control farms were more likely to have workers who used showers when entering the selected barn (26% vs. 11%; *p =* 0.04) and washed hands or used hand sanitizer before entering the selected barn (91% vs. 83%; *p* = 0.19).

During the 14-day reference period, the use of and sharing of vehicles, including company trucks or trailers, feed trucks, and bird delivery vehicles, or equipment, such as gates/panels and skid-steer loaders, was also not associated with case status. For case and control farms, vehicles and equipment were not shared frequently.

#### 3.1.4. Dead bird disposal

Use of rendering for dead bird disposal was more common on case farms than on control farms (28% vs. 14%; *p* = 0.08; Table 4). On the other hand, incineration (17% vs. 6%; *p* = 0.09) and use of a landfill (7% vs. too few to report; *p* = 0.19) as dead bird disposal methods were more common on control farms than case farms. Case farms were more likely to have visits from vehicles used for rendering than control farms (32% vs. 19%; *p* = 0.14).

#### 3.1.5. Variables of interest found to be non-significant

Several factors found to be risk factors in previous outbreaks were explored but not found to be significant in this study (Table 5). There was not a significant difference between case and control farms in the use of a wash station or spray area for vehicles, and no significant differences in wash station practices were reported. Barn ventilation type and percentage of time the curtains were open were also not related to case status. Approximately 97% of case and control farms had gravel or dirt roads compared to hard top/asphalt roads as the road surface on the farms that vehicles coming onto the operation drive on. Use of landscape fabric (weed barrier) on curtains or air inlets, a relatively new practice, was also not found to be associated with case status.

### 3.2. Multivariable analysis

The 20 variables that passed the univariate screening, having Fisher’s exact test *p* ≤ 0.20, and were biologically plausible included the following. All variables a-s were categorical; the last variable t, was discrete numeric.

- Farm being in a control zone.
- Having both brooder and grower production on the farm.
- Having market toms on the farm.
- Having any other poultry on the farm.
- Farm was independent (vs. being a company, contract, or other type of farm).
- Water treatments (such as chlorination) given in poultry drinking water continuously (vs. intermittently or not at all).
- Wetland or swamp was within 320 meters (350 yards) of the farm.
- Any wild waterfowl or shorebirds seen on water bodies within 320 meters (350 yards) of the farm.
- Any wild waterfowl or shorebirds seen on the closest body of water to the farm.
- Any wild waterfowl or shorebirds seen on the closest crop field to the farm.
- Any wild waterfowl seen on the farm within approximately 91.4 meters (100 yards) of the outside of the barns.
- Any wild mammals (e.g., raccoons, opossums, skunks, coyotes, or foxes) or evidence of their presence seen in or around poultry barns.
- Workers shower before entering the selected barn.
- Workers wash their hands or use hand sanitizer before entering the selected barn.
- Use of a visitor log to record visitor traffic onto the farm.
- Availability of a restroom facility (including portable) for crews that visit the farm.
- Use of rendering as a dead bird (daily mortality) disposal method.
- Use of incineration as a dead bird (daily mortality) disposal method.
- Use of landfill as a dead bird (daily mortality) disposal method.
- The general weekly number of vehicles (including employee vehicles) that entered the farm (coming near the barns or not).

To avoid collinearity, a single variable (j) from the cluster of g–k was selected and offered into the multivariable models to represent wild bird exposure, and rendering (q) was selected from the list of daily mortality disposal methods because it was a risk factor [incineration (r) and landfill (s) were protective, see Supplementary material].

In the final model, imputation was only used for 2 variables: worker biosecurity includes shower before entering the barn and render dead birds. These variables were missing 2 and 3 responses out of 125 (2%), respectively, and were divided between cases and controls.

Seven variables remained in the final multivariable model (Table 6). Farms within an existing control zone had increased odds of being a case [odds ratio (OR) = 3.68, 95% confidence interval (CI) = 1.06–12.74]. Other factors associated with increased odds of H5N1 HPAI infection included having both brooder and grower turkey production on the farm (OR = 7.35, CI = 2.51–21.54) and having toms as the sex market type on the farm (OR = 6.86, CI = 1.83–25.79). Seeing wild waterfowl or shorebirds in the closest field was also associated with increased odds of infection (OR = 6.02, CI = 1.83–19.78). The use of rendering for dead bird disposal during the 14-day reference period was associated with increased odds of infection (OR = 8.26, CI = 2.25–30.34). Factors found to have a protective effect included workers entering the selected barn using a shower during the 14-day reference period at least some of the time (OR = 0.29, CI = 0.09–0.98) and having a restroom facility available to crews who visit the farm (OR = 0.32, CI = 0.10–1.05).

**Table 6:** Results of multivariable logistic regression analysis of factors associated with HPAI H5N1 infection on U.S. commercial meat turkey farms.

Characteristic	% Case farms	% Control farms	Odds ratio (95% CI)	*p*-value
In an existing control zone	31.8	11.9	3.68 (1.06–12.74)	0.04
Both brooder and grower stages on farm	51.5	27.1	7.35 (2.51–21.54)	<0.01
Sex: toms	86.4	67.8	6.86 (1.83–25.79)	<0.01
Waterfowl/shorebirds seen in closest field	30.3	11.9	6.02 (1.83–19.78)	<0.01
Worker biosecurity includes shower before entering barn^A^	10.6	27.1	0.29 (0.09–0.98)	0.05
Restroom facility available to crews visiting farm	45.5	69.5	0.32 (0.10–1.05)^B^	0.05
Render dead birds	30.3	13.6	8.26 (2.25–30.34)	<0.01

A Workers always, most of the time, or sometimes showered vs. never or shower not available. This question was asked specifically for the selected barn.

B Odds ratio is for comparison between always/sometimes available vs. never available.

Biologically plausible, first-order interactions were assessed but were not significant. A region variable (east, central, west) was offered for inclusion in the final model as a fixed effect to test for confounding by region, but no confounding was seen, so it was excluded from the final model.

A multivariate model based on data from the subset of farms linked to wild bird introductions was similar to the risk factors identified from the farm-level model described above, other than the control zone becoming non-significant (data not shown).

## 4. Discussion

The United States has experienced an unprecedented outbreak of HPAI H5N1 beginning in late 2021, with the first detections of Eurasian H5 2.3.4.4b GsGD in wild birds and followed by the first confirmed infected commercial poultry premises in early 2022. Subsequently, this outbreak has resulted in the loss of millions of commercial and backyard poultry and detections in many species of wild birds and wild mammals across the country, in addition to having severe financial consequences. With the ongoing global circulation of AIVs that have repeatedly caused large outbreaks, there remains a need to identify actions that may be helpful to prevent infection on farms (4, 24, 25). The case-control study presented here investigated the risk factors associated with infection with HPAI virus between February and October 2022 on U.S. meat turkey farms.

Our results indicated that being inside a control zone increased the odds of a farm being infected with HPAI. Proximity of a farm to the nearest infected farm was a risk factor for HPAI infection in outbreaks in Europe and Japan (26–29); and the most significant risk factor for infection on table egg layer farms during the 2014–2015 HPAI H5N2 Midwestern U.S. outbreak was the farm being located within an existing control zone (16). When analyzing the subset of data from farms likely infected by independent wild bird introductions, however, control zone did not remain in the final model. This finding may highlight the importance and effectiveness of control measures implemented inside of control zones, including rapid depopulation following detection. These measures may have minimized transmission by lateral spread, a spread mechanism implicated in a much smaller percentage of cases during 2022. Overall, our findings corroborate the importance of biosecurity and surveillance for farms located in close proximity to an infected farm to prevent infection and ensure rapid detection.

Other factors associated with HPAI infection were related to the stages of production on the farm and sex of birds. Case farms were more likely to raise toms and were more likely to have both brooder and grower stages on farm. Age of birds has been shown to impact susceptibility to virus (30, 31). Toms are typically grown several weeks longer than hens prior to movement to slaughter, and production practices, such as changes in ventilation, may differ during those extra weeks that toms are on farm. The increased age and additional time on farm, which includes further exposures to fomites such as personnel and vehicles, may account for the increased risk of case status for farms with toms. Similarly, farms with birds of differing ages, such as brooders and growers, may be uniquely susceptible. Although production stage and sex of birds raised on a particular farm may not be easily changed due to the structure of the poultry industry, this information regarding risk could be used to inform surveillance activities and guide the implementation of increased biosecurity on farms raising toms and multiple stages of production.

Rendering as a method of dead bird disposal for normal daily mortality during the 14-day reference period was also a risk factor for infection. Various methods of dead bird disposal are used by poultry farms, including on-farm approaches, such as burial and composting, and off-farm approaches, such as rendering and landfill. Rendering requires the regular removal of dead bird carcasses from the farm and movement to a renderer, where carcasses are converted to useable by-products. Rendering has been reported as an important risk factor in previous AIV outbreaks (16, 17, 32–34). Movement of virus in carcasses and feathers from a farm to the renderer prior to detection and vehicle movements are possible modes of transmission. We found that rendering vehicles coming onto the farm vs. not coming to the farm at all was significant (*p* = 0.14) in the univariate analysis. We also asked several follow-up questions to respondents using rendering on farm to better understand the risk of this practice, including covering the carcass bin, means of transport, and how frequently carcasses are moved to the renderer; however, none of these variables were significant in the analysis. Interestingly, when data were analyzed by introduction route, rendering remained a risk factor for infection even when analyzing only farms infected as a result of independent wild bird introductions. Given this finding and the continued finding of rendering as a risk factor in multiple outbreaks, dead bird disposal practices should be investigated further. Future studies should consider adding more detailed questions to identify specific risk factors and protective factors for all methods of dead bird disposal, not just rendering. For example, if disposal is on-farm vs. off-farm, if methods are shared with other farms, how carcasses are moved from the barn to either a holding area or to disposal, whether carcass handling attracts wildlife or wild birds, frequency of movement to disposal, and any equipment or vehicles used in association with rendering and their disinfection.

Two biosecurity measures remained in the final model and were found to be protective: workers having access to and using a shower at least some of the time when entering the selected barn and having a restroom facility (including portable) available to crews that visit the farm. No follow-up questions were asked about this management practice, but the availability of a restroom may improve hand hygiene and reduce human movements on the farm, particularly movements in and around barns and surrounding areas. Although not retained in the final model, workers on control farms were also more likely to wash hands or use hand sanitizer before entering the barn. Contaminated fomites, such as hands and clothing, can contribute to viral spread (6, 29, 35). Worker shower use, hand washing, and the availability of a restroom are biosecurity practices that can help reduce the indirect transmission of virus into poultry barns. The importance of on-farm biosecurity has been previously highlighted, especially for personnel moving between poultry farms and for those not only coming onto poultry farms but also entering poultry barns (16, 17, 29, 34, 35). Providing restroom facilities could improve hygiene measures and restrict human movements and barn entry to only those barns or areas where work is being performed. Implementing on-farm biosecurity measures for workers and visitors is an essential component of disease prevention, and these findings may be considered in the development of farm biosecurity plans.

Phylogenetic analyses indicated that independent wild bird introductions were the predominant route of introduction of virus onto turkey farms in the U.S. in 2022. This is in contrast to the 2014–2015 outbreak, which was predominated by lateral (farm-to-farm) transmission (5, 13). Most introductions are likely due to indirect contact with wild birds or undefined mechanisms, although direct contact cannot be ruled out, particularly in instances where birds have access to the outdoors and there is a possibility of mingling with wild birds (6, 36–39). Case farms were more likely to report observations of wild birds in proximity to farms and nearby fields and waterbodies, and to report wild bird habitat such as wetlands or swamps within 320 meters (350 yards) of the farm. Farm proximity to water and wild bird habitat, as well as presence of high densities of migratory wild waterfowl, have been identified as risk factors in previous outbreaks (37, 38, 40, 41). Concentrations of domestic poultry in combination with high densities of wild birds provides a potential interface for viral transmission and spill-over events; and necessitates the identification and implementation of protective biosecurity measures to limit introduction. When data from this case-control study were analyzed by sub-setting those farms likely infected by wild bird introductions, few changes were observed in the risk or protective factors for being a case farm. The similarities between the full and sub-set models are likely explained by the predominance of wild bird introductions in the full dataset. Although some factors, for example, farm location near bodies of water, cannot be changed, measures can be undertaken to mitigate the possibility of associated direct or indirect exposures. We asked some questions about measures taken to minimize wildlife and wild bird activity on-farm and entry into barns, but given the important role of wild birds in the dynamics of the U.S. HPAI H5N1 outbreak, additional work to explore on-farm protective measures is needed. Practices such as reducing water pooling, minimizing wildlife attractants and food sources, using trained dogs, implementing laser technology, and using decoys have been used to prevent direct and indirect contacts at the interface of wild birds and poultry. Additional studies to elucidate the utilization and effectiveness of these practices would be useful (42–44).

It is important to acknowledge the limitations of this study. Recall bias is a consideration for data collection via surveys. Respondents in the current study were asked to provide responses for activities and observations that had taken place an average of 7 months prior, with the difference between the date of the interview and the beginning of the reference period as little as 2 months and as long as 12 months prior. It is also possible that recall and observations may have been different for case farms vs. control farms, as case farms may have been more likely to reflect on the time period prior to detection of infection. Another limitation of survey methodology is bias associated with questions that may be considered sensitive, providing responses that may be considered more favorable or that follow biosecurity plans vs. being reflective of actual practices. While attempts were made to balance the numbers of completed case and control questionnaires geographically, it was not possible to perfectly match cases and controls by state due to non-response and, in some situations, a lack of sufficient control farms. Finally, the results of this study are representative of U.S. production practices and of the viral dynamics of the 2022 HPAI H5N1 outbreak and, therefore, may not be directly applicable to production systems in other countries or future outbreaks with different viruses.

Future work may help further improve our understanding of the complex epidemiology of avian influenza transmission at the interface of wild birds and domestic poultry. Two additional topics were included in the case-control questionnaire but were not reported here. One section of the questionnaire was related to biosecurity investments, including questions regarding ongoing biosecurity expenses and permanent and temporary improvements made since 2015 that impact farm biosecurity. These data will be analyzed and reported separately to identify priority areas for investment in biosecurity measures to reduce risk for HPAI. The questionnaire also included challenge-level questions asking for producers’ opinions on level of challenge of certain topics, including biosecurity-, personnel-, and equipment-related challenges. Finally, weather conditions and patterns related to AIV transmission have been examined previously and could have played a role in the outbreak in 2022 (45–47). Future work could expand upon the case-control study presented here to incorporate historical weather data such as temperature, relative humidity, precipitation, and wind speed in the time preceding detection to investigate the role of weather on risk of HPAI infection.

## 5. Conclusion

This study compared management and biosecurity factors on case and control meat turkey farms in the U.S. during the HPAI H5N1 outbreak in 2022. Knowledge of risk factors for infection has become increasingly important as this outbreak continues into 2023 and as additional domestic poultry flocks, wild birds, and wildlife species are detected. Study results identified the following key risk factors: location of farms within an existing control zone, multiple stages of production on farm, toms as the sex market type on farm, waterfowl/shorebirds seen in the closest field, gaps in worker biosecurity measures such as lack of availability of a shower before entering the barn or a restroom facility for visiting crews, and the use of rendering for dead bird disposal. These risk factors were found to be associated with HPAI infection on farms and provide information that can be directly applied to support science-based updates to prevention and control recommendations to safeguard turkey farms in the United States.

## Data availability statement

The datasets presented in this article are not readily available because the data are protected from release under the Confidential Information Protection and Statistical Efficiency Act (CIPSEA, P.L. 115-435, Title III). Anonymized data may be made available upon request, for statistical purposes only, and completion of non-disclosure training, forms, and review of any output products. Requests to access the datasets should be directed to KP.

## Author contributions

VF, KP, AB, AG, MB, AF, AD, and KM conceptualized the research and study design. KP, VF, AB, and AG designed the questionnaire. RM, MB, AB, VF, and KP supported data collection. MT and KL led diagnostic sampling and interpretation of results. VF, KP, AB, RM, MB, and AG participated in the data analysis and visualization. KP and VF wrote the primary draft of the manuscript. KP, VF, AB, RM, AG, MB, AD, KM, and MT contributed to reviewing and editing the final manuscript. All authors contributed to the article and approved the submitted version.

## Funding

Data collection was funded through an interagency agreement between the National Agricultural Statistics Service (NASS) and the Animal and Plant Health Inspection Service (APHIS).

## Conflict of interest

The authors declare that the research was conducted in the absence of any commercial or financial relationships that could be construed as a potential conflict of interest.

## Publisher’s note

All claims expressed in this article are solely those of the authors and do not necessarily represent those of their affiliated organizations, or those of the publisher, the editors and the reviewers. Any product that may be evaluated in this article, or claim that may be made by its manufacturer, is not guaranteed or endorsed by the publisher.

## Footnotes

^1^ https://github.com/USDA-VS/vSNP

## Abbreviations

AIV: Avian influenza virus
CI: Confidence interval
HPAI: Highly pathogenic avian influenza
OR: Odds ratio
USDA: United States Department of Agriculture
