# Erratum: Investigation of risk factors for introduction of highly pathogenic avian influenza H5N1 infection among commercial turkey operations in the United States, 2022: a case-control study

**DOI:** 10.3389/fvets.2023.1336351

**Published:** 2023-11-23

**Authors:** Frontiers Production Office

**Affiliations:** Frontiers Media SA, Lausanne, Switzerland

**Keywords:** avian influenza, biosecurity, case control, H5N1, highly pathogenic avian influenza, risk factors, turkey

Due to a production error, there was a mistake in Table 3 as published. “Visitor log used” and “Restroom facility available to crews visiting farm” should have been in different rows.

“Visitor log” should have had levels of “Always” and “Sometimes/never”.

“Restroom facility available to crews visiting farm” should have had levels “Always/sometimes”, “Never”, “Unknown (skipped question).”

The corrected Table 3 appears below.

**Table 3:** Univariate analyses of factors (*p* ≤ 0.20) considered for entry into the multivariable model.

**Biosecurity practice**	**Level**	**Case farms (*N*/%)**	**Control farms (*N*/%)**	**Univariate *p*-value**
Workers showered^A^		7 (10.8)	15 (25.9)	0.04
Workers washed hands or used hand sanitizer^A^		54 (83.1)	53 (91.4)	0.19
Visitor log used	Always	39 (60.0)	45 (77.6)	0.05
	Sometimes/never	26 (40.0)	13 (22.4)	
Restroom facility available to crews visiting farm	Always/sometimes	30 (45.5)	41 (69.5)	0.02
	Never	26 (39.4)	12 (20.3)	
	Unknown (skipped question)	10 (15.2)	6 (10.2)	

Percentage of case farms and percentage of control farms by biosecurity practices.

^A^Workers always, most of the time, or sometimes used the practice before entering the barn vs. never or not available.

This question was asked specifically for the selected barn and for the 14-day reference period.

Due to a production error, the word “production” was misspelled as “productteion”.

A correction has been made to the section **Conclusion**, Paragraph Number: 1.

“This study compared management and biosecurity factors on case and control meat turkey farms in the U.S. during the HPAI H5N1 outbreak in 2022. Knowledge of risk factors for infection has become increasingly important as this outbreak continues into 2023 and as additional domestic poultry flocks, wild birds, and wildlife species are detected. Study results identified the following key risk factors: location of farms within an existing control zone, multiple stages of production on farm, toms as the sex market type on farm, waterfowl/shorebirds seen in the closest field, gaps in worker biosecurity measures such as lack of availability of a shower before entering the barn or a restroom facility for visiting crews, and the use of rendering for dead bird disposal. These risk factors were found to be associated with HPAI infection on farms and provide information that can be directly applied to support science-based updates to prevention and control recommendations to safeguard turkey farms in the United States.”

The publisher apologizes for these mistakes. The original article has been updated.

