# Supplementary material for: Investigation of risk factors for introduction of highly pathogenic avian influenza H5N1 infection among commercial turkey operations in the United States, 2022: a case-control study
Source: Front Vet Sci. 2023 Aug 30;10:1229071. doi: 10.3389/fvets.2023.1229071 (PMC10498466; doi:10.3389/fvets.2023.1229071)
Supplement: Supplementary file 1 [file Data_Sheet_1.pdf]

UNITED STATES DEPARTMENT OF AGRICULTURE<br>ANIMAL AND PLANT HEALTH INSPECTION SERVICE<br>VETERINARY SERVICES<br>2150 CENTRE AVE, BLDG B<br>FORT COLLINS, CO 80526	**COMMERCIAL TURKEY CASE CONTROL SURVEY**

According to the Paperwork Reduction Act of 1995, an agency may not conduct or sponsor, and a person is not required to respond to a collection of information unless it displays a valid OMB control number. The valid OMB control number for this information collection is 0579-0484. The time required to complete this information collection is estimated to average 75 minutes per response, including the time to review instructions, search existing data resources, gather the data needed, and complete and review the information collected.	OMB Approved<br>0579-0484<br><br>EXP: 04/30/2023

The information you provide will be used for statistical purposes only. Your responses will be kept confidential and any person who willfully discloses ANY identifiable information about you or your operation is subject to a jail term, a fine, or both. This survey is conducted in accordance with the Confidential Information Protection and Statistical Efficiency Act of 2018, Title III of Pub. L. No. 115-435, codified in 44 U.S.C. Ch. 35 and other applicable Federal laws. For more information on how we protect your information please visit: https://www.nass.usda.gov/confidentiality. Response is **voluntary**.

Date (mm/dd/yy): _______________ date

Section A – Case or Control

1. Is this a case or control farm? **[During the interview, verify the farm name and address printed on the label are correct for this farm.]** t101

   ☐1 Case farm **[Go to Item 2.]**

   ☐3 Control farm **[Go to Item 3.]**

2. If this is a **case** farm,
   a. The following is the 14-day reference period for this farm:

      t102/t102a ________________ – ________________ mm/dd/yy – mm/dd/yy

In this questionnaire, we will ask many questions about a 14-day reference period. The "reference period" for your farm is the 14 days between [Insert the dates listed in Item 2a above]. This is the 14 days before the detection of HPAI on this farm.

   b. How many turkeys were on this farm on the last day of the reference period? t103______ # turkeys
   c. During the 2022 HPAI outbreak, how many of the barns on this farm were confirmed or were suspected to be infected with HPAI? .... t104 __________# barns

[Go to Section B.]

3. If this is a **control** farm,
   a. The tentative 14-day reference period for this farm is:

      t105/t105a ________________ – ________________ mm/dd/yy – mm/dd/yy

   b. Did you have turkeys for the entire 14-day period between the dates in Item 3a above? t106

      ☐1 Yes

      ☐3 No

If Yes, the "14-day reference period" for your farm is [Insert the dates listed in Item 3a above]. We will refer to this as the "reference period" throughout the questionnaire. *[Proceed to Item 3c.]*

If No, help the producer identify the closest 14-day period to the reference period from Item 3a during which they had turkeys on the farm and enter that period into the fields below. This period must be during 2022.

Enter the selected 14-day period here:	t107 Start date ____________ mm/dd/yy
(Finish date = start date + 14 days)	t108 Finish date ___________ mm/dd/yy

All questions regarding the "reference period" refer to the 14 days selected above. We will refer to this as the "reference period" throughout the questionnaire.

[If the farm did not have turkeys during 2022, go to Section L.]

   c. How many turkeys were on this farm on the last day of the reference period? t109______ # turkeys

Section B – Premises Description

1. What stage(s) of turkey production is on this farm?
   a. Brooder..t201 ☐1 Yes ☐3 No
   b. Grower ....t202 ☐1 Yes ☐3 No
   c. Breeder..t203 ☐1 Yes ☐3 No
   d. Other (specify:____________________) t204oth ....t204 ☐1 Yes ☐3 No

2. What is the sex of the market type on this farm? *[Check all that apply.]*
   ☐1 Hens t205
   ☐1 Toms t206
   ☐1 Breeder hens t207
   ☐1 Breeder toms t208

3. Is this farm multiple age or single age? *[Check one only.]* t209
   ☐1 Multiple age
   ☐2 Single age

4. What other type(s) of poultry is present on this farm?
   a. Broiler ....t210 ☐1 Yes ☐3 No
   b. Layer ....t211 ☐1 Yes ☐3 No
   c. Domestic ducks or geese (exclude wild birds) ....t212 ☐1 Yes ☐3 No
   d. Other (specify:____________________) t213oth ....t213 ☐1 Yes ☐3 No

5. Is this farm certified organic?..t214 ☐1 Yes ☐3 No

6. Is this facility enrolled in NPIP?..t215 ☐1 Yes ☐3 No
   a. If yes, is this facility enrolled in an NPIP Avian Influenza Program?.........t216 ☐1 Yes ☐3 No

7. Is this a: *[Check one only.]* t217
   ☐1 Company farm?
   ☐2 Contract farm?
   ☐3 Independent farm?
   ☐4 Other? (specify: ____________________________) t217oth

8. How many barns are on this farm?.. t218_________ # barns
   a. In the last year, how many of these barns housed birds?.................................... t219_________ # barns

For the remainder of the questionnaire, some questions will ask about practices for the entire farm, and other questions will ask about practices for a "selected barn."

INSTRUCTIONS for selecting a barn:

Case farm: Select the **first** barn on this premises that was confirmed to be HPAI positive. If more than one barn was confirmed to be HPAI positive on the same date, choose one barn. **Answer questions for the 14 days prior to the onset of clinical signs or increased mortality (the reference period). [Section A, Item 2a]**

Control farm: Randomly select one barn to be the "**selected barn**." Choose one that had birds during the 14-day reference period [Section A, Item 3]. Use this barn to answer all questions about the "**selected barn**."

9. What is the barn ID or name for the **selected barn**? ....t220 _________________

10. During the 14-day reference period, did any birds on the farm or **selected barn** have access to the outdoors?
    a. Any birds on the farm ....t221 ☐1 Yes ☐3 No
    b. **Selected barn** ....t222 ☐1 Yes ☐3 No

11. Were any livestock, excluding poultry, on the farm, or located within 350 yards of the farm, fed hay or grain in the pasture or in outdoor feed troughs during the 14-day reference period? (350 yards is about the length of three football fields.) ....t223 ☐1 Yes ☐3 No ☐4 Don't Know

12. What is the water source for poultry?
    a. Off-site fresh water (for example, municipal, federal, cooperative, community, commercial) ....t224 ☐1 Yes ☐3 No
    b. Well................................t225 ☐1 Yes ☐3 No
    c. Surface water (for example, pond)................................t226 ☐1 Yes ☐3 No
    d. Other (specify:_____________________) t228oth ....t227 ☐1 Yes ☐3 No

13. Are water treatments such as chlorination used in the drinking water for the poultry on this farm? t228 ☐1 Yes ☐3 No
    a. If Yes, are these treatments given: ....t229 ☐1 Continuously? ☐3 Intermittently?

14. Are windbreaks present on this farm?

Windbreak type	Present?	If Yes, distance to closest poultry barn	
a. Evergreen or juniper	☐1 Yes ☐3 No	_______ yards	t230/t233
b. Deciduous tree	☐1 Yes ☐3 No	_______ yards	t231/t234

c. Structural (for example, hill, natural break)	☐1 Yes ☐3 No	________ yards	t232/t235

15. Are the following water body type(s) visible or within 350 yards (about three football fields) of this farm?
   - a. Pond .... t236 ☐1 Yes ☐3 No
   - b. Lake .... t237 ☐1 Yes ☐3 No
   - c. Stream .... t238 ☐1 Yes ☐3 No
   - d. River .... t239 ☐1 Yes ☐3 No
   - e. Wetland or swamp .... t240 ☐1 Yes ☐3 No
   - f. Wastewater lagoon .... t241 ☐1 Yes ☐3 No
   - g. Standing water during the 14-day reference period .... t242 ☐1 Yes ☐3 No
   - h. Drainage ditch or canal .... t243 ☐1 Yes ☐3 No
   - i. Other (specify:______________________) t245oth .... t244 ☐1 Yes ☐3 No

[If Question 15 a through i are all equal to No, skip to Question 17.]

16. For those water bodies, including drainage ditches and lagoons within 350 yards on the farm, approximately how many wild waterfowl or shorebirds (for example, ducks, geese, wading birds, gulls) were seen on the water during the 14-day reference period? *[Check one only.]* t245

   ☐1 None ☐2 Tens ☐3 Hundreds ☐4 Thousands ☐5 Don't know

17. What is the distance (in yards) of the closest body of water (for example, pond, lake, stream, river, wetland) to this farm? .... t246 ____________ yards

18. In the 14-day reference period, approximately how many wild waterfowl or shorebirds (for example, ducks, geese, wading birds, gulls) might have been seen on this body of water at one time? *[Check one only.]* t247

   ☐1 None ☐2 Tens ☐3 Hundreds ☐4 Thousands ☐5 Don't know

19. What is the approximate distance (in yards) to the closest field where crops or hay are harvested?

   t248 ____________ yards

20. What crop was last grown in this field? *[Check one only.]* t249

   ☐1 Corn

   ☐2 Soybeans

   ☐3 Alfalfa or grass intended for livestock feed

   ☐4 Other (specify: ________________________________) t250oth

   ☐5 Don't know

21. Was this field tilled in:

a. Fall 2021? .... t250 ☐1 Yes ☐3 No ☐4 Don't know

b. Spring 2022? .... t251 ☐1 Yes ☐3 No ☐4 Don't know

22. Was this field actively worked (for example, tilled, disked, hay harvested, trees cut, row crops harvested) during the 14-day reference period? .... t252 ☐1 Yes ☐3 No ☐4 Don't know

23. For this closest field, approximately how many wild waterfowl or shorebirds (for example, ducks, geese, wading birds, gulls) were seen during the 14-day reference period? *[Check one only.]* t253

☐1 None ☐2 Tens ☐3 Hundreds ☐4 Thousands ☐5 Don't know

Section C – Wild Birds

1. During the 14-day reference period, how frequently were the following types of wild birds seen on the farm and within 100 yards of the outside of the barns?

Bird type	Often	Sometimes	Never	
a. Waterfowl (for example, ducks, geese)	☐1	☐2	☐3	t301
b. Gulls	☐1	☐2	☐3	t302
c. Small perching birds (for example, sparrows, starlings, swallows)	☐1	☐2	☐3	t303
d. Blackbirds and crows	☐1	☐2	☐3	t304
e. Other water birds (for example, egrets, cormorants)	☐1	☐2	☐3	t305
f. Wild turkeys, pheasants, quail	☐1	☐2	☐3	t306
g. Raptors (for example, eagles, hawks, owls, vultures)	☐1	☐2	☐3	t307
h. Pigeons and doves	☐1	☐2	☐3	t308
i. Other (specify: _______________) t309oth	☐1	☐2	☐3	t309

2. During the 14-day reference period, how frequently were the following types of wild birds seen inside the **selected barn**?

Bird type	Often	Sometimes	Never	
a. Large birds (for example, pigeons, crows)	☐1	☐2	☐3	t310

b. Small birds (for example, finches, sparrows, starlings)	☐1	☐2	☐3	t311
c. Other (specify: _______________) t312oth	☐1	☐2	☐3	t312

3. During the 14-day reference period, did you or other farm workers observe any of the following types of **sick or dead** wild birds **inside** the barns or **outside** of the barns?

**Sick/dead bird type**	**Inside the barns**	**Outside the barns**	
a. Large birds (for example, pigeons, crows)	☐1 Yes ☐3 No	☐1 Yes ☐3 No	t313/t316
b. Small birds (for example, finches, sparrows, starlings)	☐1 Yes ☐3 No	☐1 Yes ☐3 No	t314/t317
c. Other (specify: _______________) t315oth	☐1 Yes ☐3 No	☐1 Yes ☐3 No	t315/t318

4. If Yes to Questions 3a, 3b, or 3c, what was done with the sick or dead wild birds?
   a. Left for predators ....t319 ☐1 Yes ☐3 No
   b. Disposal by same method used for daily turkey mortality on farm ....t320 ☐1 Yes ☐3 No
   c. Taken to rehab center, animal control or veterinarian............................t321 ☐1 Yes ☐3 No
   d. Something else (specify: __________________) t322oth ....t322 ☐1 Yes ☐3 No

Section D – Farm Biosecurity

1. What best describes the road surface on this farm that vehicles coming onto the operation drive on? *[Check one only.]* t401
   ☐1 Hard top/asphalt
   ☐2 Gravel
   ☐3 Dirt
   ☐4 Other (specify: ______________________________) t401oth

2. In general, do the following types of vehicles:

**Codes for question 2**
1 = come to the perimeter of the farm only
2 = enter the farm but not near the barns
3 = come near the barns
4 = do not come at all

Enter the codes that apply.

a. Garbage or dumpster pick up...t402 _____ code

b. Propane delivery...t403 _____ code

c. Feed delivery ....t404 _____ code

d. Feed ingredient delivery...t405 _____ code

e. Renderer...t406 _____ code

f. Company personnel (for example, catch/vaccination crew, barn workers, service person, veterinarian)...t407 _____ code

g. Other business visitors (for example, meter reader, repairman).............t408 _____ code

3. In general, how many vehicles (including employee vehicles) come to the following locations on a weekly basis?

a. Perimeter of the farm only .... t409 _______vehicles per week

b. Enter the farm but not near the barns.................................... t410 _______vehicles per week

c. Come near the barns .... t411 _______vehicles per week

4. Excluding driveways on farm, what is the distance (in yards or miles) from this farm to the nearest public gravel or dirt road? ....t412y ________ yards OR t412m __________ miles

5. How frequently is vegetation mowed and/or bush hogged on the premises? (Answer for when vegetation is present, for example, spring and summer) ....t413 ______ times/month

6. Was there a wash station or spray area being used for vehicles during the 14-day reference period?

t414 ☐1 Yes ☐3 No

[If Question 6 = No, SKIP to Question 8.]

7. During the 14-day reference period, was the vehicle wash station or spray area:

a. Located on the farm?...t415 ☐1 Yes ☐3 No

b. Were the vehicle tires washed?...t416 ☐1 Yes ☐3 No

c. Was the vehicle exterior washed? ....t417 ☐1 Yes ☐3 No

d. Was the vehicle interior cleaned (for example, floor mats) ....t418 ☐1 Yes ☐3 No

e. Were the following vehicles washed?

i. Worker vehicles ....t419 ☐1 Yes ☐3 No ☐4 NA

ii. Feed trucks ....t420 ☐1 Yes ☐3 No ☐4 NA

iii. Vehicles delivering or removing birds ....t421 ☐1 Yes ☐3 No ☐4 NA

iv. Other vehicles (specify: ________________) t422oth ....t422 ☐1 Yes ☐3 No ☐4 NA

f. What disinfectant was used? .... t423 ______________________

g. What was the distance from the vehicle wash station to the **selected barn** in yards?

t424 _________yards

8. Did workers and visitors always, sometimes, or never park in a restricted area away from the poultry barns during the 14-day reference period?
   a. Workers..t425 ☐1 Always ☐2 Sometimes ☐3 Never
   b. Visitors..t426 ☐1 Always ☐2 Sometimes ☐3 Never

9. During the 14-day reference period, were wild mammals, such as raccoons, opossums, skunks, coyotes, or foxes, or evidence of their presence, seen in or around poultry barns?
   t427 ☐1 Yes ☐3 No

10. During the 14-day reference period, which of the following pest and wild bird control measures were used on this farm?
   a. Rat and mouse bait stations ....t428 ☐1 Yes ☐3 No
      i. If Yes, how frequently are they checked per month? ....t429 _______ times/month
   b. Beetle control (for example, sprays, baits, boric acid) ....t430 ☐1 Yes ☐3 No
   c. Fly control (for example, baits, larvicide, space sprays/fogger, biological predators)
      ....t431 ☐1 Yes ☐3 No
   d. Netting on barns to prevent wild bird access.......................................t432 ☐1 Yes ☐3 No

11. How often were rodents observed in the **selected barn** during the 14-day reference period? *[Check one only.]* t433
   ☐1 Frequently (for example, daily)
   ☐2 Occasionally (for example, weekly)
   ☐3 Never

12. What was the intensity of beetles observed in the **selected barn** during the 14-day reference period? *[Check one only.]* t434
   ☐1 High
   ☐2 Medium
   ☐3 Low
   ☐4 None

13. What was the intensity of flies observed in the **selected barn** during the 14-day reference period? *[Check one only.]* t435
   ☐1 High
   ☐2 Medium
   ☐3 Low
   ☐4 None

14. Does the **selected barn** have a hard-surface entry pad (for example, concrete, asphalt)?

t436 ☐1 Yes ☐3 No

If Yes,

a. Is the entry pad cleaned?..t437 ☐1 Yes ☐3 No

i. If Yes, specify frequency t438/t438a ______times/ ☐1 week ☐2 month OR ☐3 year

b. Is disinfectant used?..t439 ☐1 Yes ☐3 No

15. During the 14-day reference period, how frequently were wild birds, wild animals, and rodents able to access poultry feed or feed ingredients (for example, feed spillage, open bag, cover left open)?

For this question, "Always" is 100% of the time, "Most of the time" is 51-99% of the time, "Sometimes" is 1-50% of the time, and "Never" is 0% of the time.

Type	Always	Most of the time	Sometimes	Never	
a. Wild birds	☐1	☐2	☐3	☐4	t440
b. Wild animals (such as raccoons, opossums, skunks, coyotes, or foxes)	☐1	☐2	☐3	☐4	t441
c. Rodents	☐1	☐2	☐3	☐4	t442

16. Does this farm have a written wildlife management plan that includes methods to minimize wildlife or wild bird entry and reduce wildlife attractants such as standing water? t443 ☐1 Yes ☐3 No

17. In the 2 years before the 14-day reference period, were any biosecurity audits or assessments (company or third party) conducted on this farm? t444 ☐1 Yes ☐3 No ☐4 Don't know

18. Considering the following biosecurity topics, how challenging would you say these are for producers to achieve? *[Check one box per row.]*

	Not at all challenging	Slightly challenging	Somewhat challenging	Quite challenging	Extremely challenging	
a. Keeping feed safe from rodents	☐1	☐2	☐3	☐4	☐5	t445
b. Keeping feed safe from wildlife	☐1	☐2	☐3	☐4	☐5	t446

Section E – Biosecurity Investments

1. Over the past year, has this farm had the following ongoing biosecurity expenses?
   If Yes, what was the typical monthly cost of each?

Biosecurity type	Ongoing expenses?	If Yes, what is the typical monthly cost?	
a. Wash station or spray area being used for vehicles	☐1 Yes ☐3 No	$________	t501/t507
b. Foot baths	☐1 Yes ☐3 No	$________	t502/t508
c. Pest and bait stations	☐1 Yes ☐3 No	$________	t503/t509
d. Wash stations for employees (for example, sinks, showers)	☐1 Yes ☐3 No	$________	t504/t510
e. PPE for employees and visitors (for example, gloves, coveralls, boot covers)	☐1 Yes ☐3 No	$________	t505/t511
f. Other (specify: _______________) t506oth	☐1 Yes ☐3 No	$________	t506/t512
**Total monthly cost**		$________	t513

2. Since 2015, has this farm built or made **permanent** improvements or renovations on the following farm structures that impact the farm's biosecurity?
   a. A service room that personnel must enter through that separates "outside area" from "inside area" (for example, Danish entry) .... t514 ☐1 Yes ☐3 No
   b. Wash stations for employees (for example, sinks, showers)............ t515 ☐1 Yes ☐3 No
   c. Permanent improvements or renovations to limit wild bird access to barns t516 ☐1 Yes ☐3 No
   d. Barn ventilation system............ t517 ☐1 Yes ☐3 No
   e. Other barn improvements or renovations............ t518 ☐1 Yes ☐3 No
   f. Feed bins .... t519 ☐1 Yes ☐3 No
   g. Permanent vehicle wash stations (for example, automated truck wash)... t520 ☐1 Yes ☐3 No
   h. Other (specify: ____________________) t521oth .... t521 ☐1 Yes ☐3 No
   i. If Yes to any in 2a through 2h, what was the approximate total cost of **all** of these improvements?
      t522 $______________

3. Over the next two years, does this farm have plans to build or make **permanent** improvements or renovations on farm structures such as barns, feed bins, or other structures that impact the farm's biosecurity?............ t523 ☐1 Yes ☐3 No

4. Since 2015, has this farm built or installed any of the following **temporary** structures or infrastructure that impact the farm's biosecurity?
   a. Gates ....t524 ☐1 Yes ☐3 No
   b. Parking area...t525 ☐1 Yes ☐3 No
   c. Temporary wild bird mitigation ....t526 ☐1 Yes ☐3 No
   d. Landscape fabric on air intake inlets or curtains..................................t527 ☐1 Yes ☐3 No
   e. Temporary vehicle wash stations (for example, hand sprayer) ....t528 ☐1 Yes ☐3 No
   f. Other (specify: ____________________) t529oth ....t529 ☐1 Yes ☐3 No
   g. If Yes to any in 4a through 4f, what was the approximate total cost of **all** of these improvements?
   t530 $______________

5. Over the next two years, does this farm have plans to build or install any **temporary** structures that impact the farm's biosecurity? ....t531 ☐1 Yes ☐3 No

6. How much did the 2014-2015 HPAI outbreak influence your decisions about biosecurity investments for this farm? ....t532 ☐1 Not at all ☐2 Slightly ☐3 Somewhat ☐4 Quite a bit ☐5 Extremely

Section F – Farm Help / Workers

Questions in this section refer to persons such as the producer, employees, farm help, crews, etc.

1. What is the total number of employees working on this farm that have access to or directly work with poultry (including family, both paid and unpaid)? ....t601 ___________ #

2. Did this farm use occasional or emergency workers such as family members or part-time help to fill in for any employees during the 14-day reference period?..............................t602 ☐1 Yes ☐3 No

3. During the 14-day reference period, how frequently were the following measures used by workers entering the **selected barn**?

   For this question, "Always" is 100% of the time, "Most of the time" is 51-99% of the time, "Sometimes" is 1-50% of the time, and "Never" is 0% of the time. N/A=not applicable.

Measure	Always	Most of the time	Sometimes	Never	N/A-Not available	
a. An established clean/dirty line	☐1	☐2	☐3	☐4	☐5	t603
b. A service room that personnel must enter through that separates "outside area" from "inside area"	☐1	☐2	☐3	☐4	☐5	t604

c. Shower	☐1	☐2	☐3	☐4	☐5	t605
d. Wash hands or use hand sanitizer before entering the barn	☐1	☐2	☐3	☐4	☐5	t606
e. Wear disposable gloves	☐1	☐2	☐3	☐4	☐5	t607
f. Different personnel for different barns	☐1	☐2	☐3	☐4	☐5	t608
g. Locks on the barn doors	☐1	☐2	☐3	☐4	☐5	t609
h. Wear disposable coveralls	☐1	☐2	☐3	☐4	☐5	t610
i. Change of clothing/coveralls (washable)	☐1	☐2	☐3	☐4	☐5	t611
j. Change of shoes or use of shoe covers	☐1	☐2	☐3	☐4	☐5	t612
k. Scrub footwear (bucket and brush)	☐1	☐2	☐3	☐4	☐5	t613
l. Foot bath (liquid)	☐1	☐2	☐3	☐4	☐5	t614
m. Foot bath (dry, such as powdered or particulate)	☐1	☐2	☐3	☐4	☐5	t615

[If both Question 3 l and m = not available, SKIP to Question 5.]

4. What was the frequency that liquid or dry footbath solutions were changed for the **selected barn** during the 14-day reference period? t616/t616a _______________times/ ☐1 week ☐2 month OR ☐3 year
   a. What disinfectant was used in the footbaths? .... t617 ____________________________

5. During a typical month, do any workers on this farm visit another poultry farm?
   t618 ☐1 Yes ☐3 No ☐4 Don't know

6. Are any workers or members of their household employed by other poultry operations, other company farms, rendering plants, or processing plants?
   a. Workers.. t619 ☐1 Yes ☐3 No ☐4 Don't know
   b. Members of household .... t620 ☐1 Yes ☐3 No ☐4 Don't know

7. Do any employees own their own poultry, including small backyard flocks?
   t621 ☐1 Yes ☐3 No ☐4 Don't know

8. Are employees required to stay off farm after exposure to other poultry?......... t622 ☐1 Yes ☐3 No
   a. If Yes, for how long (hours)?.. t623 ________hours

9. In a typical week, how much time is spent by **all** employees on biosecurity activities on the farm?

t624 ________hours

10. Considering the following personnel-related topics, how challenging would you say these are for producers to achieve? *[Check one box per row.]*

	Not at all challenging	Slightly challenging	Somewhat challenging	Quite challenging	Extremely challenging	
a. Hiring new personnel	☐1	☐2	☐3	☐4	☐5	t625
b. Retaining trained personnel	☐1	☐2	☐3	☐4	☐5	t626
c. Communicating the importance of biosecurity to personnel	☐1	☐2	☐3	☐4	☐5	t627
d. Enforcing daily biosecurity measures	☐1	☐2	☐3	☐4	☐5	t628
e. Other personnel-related challenges (Specify: t629oth ________)	☐1	☐2	☐3	☐4	☐5	t629

Section G – Farm Visitors

1. How often is a visitor log used to record visitor traffic onto the farm?

t701 ☐1 Always ☐2 Sometimes ☐3 Never

2. Did any of the following types of people visit the farm during the 14-day reference period? If Yes, how many times did they visit during the 14-day reference period and did they enter the **selected barn**?

Visitor type	Did they visit the farm?	If Yes,	
		How many times did they visit?	Did this visitor enter the selected barn?

a. Federal/State veterinary or animal health worker	☐1 Yes ☐3 No	_____ # visits	☐1 Yes ☐3 No	t702/t725/t748
b. Extension agent or university veterinarian	☐1 Yes ☐3 No	_____ # visits	☐1 Yes ☐3 No	t703/t726/t749
c. Private or company veterinarian	☐1 Yes ☐3 No	_____ # visits	☐1 Yes ☐3 No	t704/t727/t750
d. Company service person	☐1 Yes ☐3 No	_____ # visits	☐1 Yes ☐3 No	t705/t728/t751
e. Nutritionist or feed company consultant	☐1 Yes ☐3 No	_____ # visits	☐1 Yes ☐3 No	t706/t729/t752
f. Bird delivery personnel (for example, poult placement, brood to grow move)	☐1 Yes ☐3 No	_____ # visits	☐1 Yes ☐3 No	t707/t730/t753
g. Vaccination crew	☐1 Yes ☐3 No	_____ # visits	☐1 Yes ☐3 No	t708/t731/t754
h. Catch crew (bird removal)	☐1 Yes ☐3 No	_____ # visits	☐1 Yes ☐3 No	t709/t732/t755
i. Artificial insemination crew (for breeder farms)	☐1 Yes ☐3 No	_____ # visits	☐1 Yes ☐3 No	t710/t733/t756
j. Feed ingredient delivery person	☐1 Yes ☐3 No	_____ # visits	☐1 Yes ☐3 No	t711/t734/t757
k. Feed delivery personnel	☐1 Yes ☐3 No	_____ # visits	☐1 Yes ☐3 No	t712/t735/t758
l. Egg truck personnel (for breeder farms)	☐1 Yes ☐3 No	_____ # visits	☐1 Yes ☐3 No	t713/t736/t759
m. Fresh litter delivery services	☐1 Yes ☐3 No	_____ # visits	☐1 Yes ☐3 No	t714/t737/t760
n. Litter removal services (for example, litter broker, litter disposal)	☐1 Yes ☐3 No	_____ # visits	☐1 Yes ☐3 No	t715/t738/t761
o. Customer (private individual)	☐1 Yes ☐3 No	_____ # visits	☐1 Yes ☐3 No	t716/t739/t762
p. Wholesaler, buyer, or dealer	☐1 Yes ☐3 No	_____ # visits	☐1 Yes ☐3 No	t717/t740/t763
q. Renderer	☐1 Yes ☐3 No	_____ # visits	☐1 Yes ☐3 No	t718/t741/t764
r. Dead bird pickup other than by renderer	☐1 Yes ☐3 No	_____ # visits	☐1 Yes ☐3 No	t719/t742/t765
s. Rodent control crew	☐1 Yes ☐3 No	_____ # visits	☐1 Yes ☐3 No	t720/t743/t766

t.	Occasional worker (for example, family member, part-time help over holiday)	☐1 Yes ☐3 No	_____ # visits	☐1 Yes ☐3 No	t721/t744/t767
u.	Construction workers, repair or maintenance personnel	☐1 Yes ☐3 No	_____ # visits	☐1 Yes ☐3 No	t722/t745/t768
v.	Other business visitors (including other producers, meter readers, package delivery (UPS), propane, or similar)	☐1 Yes ☐3 No	_____ # visits	☐1 Yes ☐3 No	t723/t746/t769
w.	Other nonbusiness visitors (including neighbors, family members, friends, and school field trips)	☐1 Yes ☐3 No	_____ # visits	☐1 Yes ☐3 No	t724/t747/t770

3. For those visitors who entered the **selected barn** during the 14-day reference period, did you require the following? *[Check one per row.]*

	**Yes, verified at farm**	**Yes, visitor responsibility**	**No**	
a. Change of outer clothing/farm specific clothing/coveralls	☐1	☐2	☐3	t771
b. Foot covers or change of footwear	☐1	☐2	☐3	t772
c. Mask	☐1	☐2	☐3	t773
d. Hand sanitizing or handwashing	☐1	☐2	☐3	t774
e. Gloves	☐1	☐2	☐3	t775
f. Not visit multiple farms in the same day	☐1	☐2	☐3	t776
g. Other (specify: _______________) t777oth	☐1	☐2	☐3	t777

4. How often is a restroom facility (including portable) available to crews that visit the farm?

t778 ☐1 Always (24 hours/day) ☐2 Sometimes ☐3 Never

Section H – Farm Vehicles and Equipment

1. Were the following vehicles shared with another farm during the 14-day reference period? If Yes, how often were they cleaned and disinfected prior to returning to this farm?

Vehicle type	Shared with another farm in the 14-day reference period?	If Yes, how often was it cleaned and disinfected prior to returning to this farm?	
a. Company trucks or trailers (for example, pickup truck, trailer with supplies, supervisor truck, or similar)	☐1 Yes ☐3 No	☐1 Always<br>☐2 Sometimes<br>☐3 Never	t801/t810
b. Feed trucks	☐1 Yes ☐3 No	☐1 Always<br>☐2 Sometimes<br>☐3 Never	t802/t811
c. Feed ingredient truck	☐1 Yes ☐3 No	☐1 Always<br>☐2 Sometimes<br>☐3 Never	t803/t812
d. Bird delivery vehicles (for example, placing birds)	☐1 Yes ☐3 No	☐1 Always<br>☐2 Sometimes<br>☐3 Never	t804/t813
e. Bird removal vehicles (for example, moved to slaughter, moved to grow)	☐1 Yes ☐3 No	☐1 Always<br>☐2 Sometimes<br>☐3 Never	t805/t814
f. Egg removal vehicles (for breeder farms)	☐1 Yes ☐3 No	☐1 Always<br>☐2 Sometimes<br>☐3 Never	t806/t815
g. Manure/litter hauling	☐1 Yes ☐3 No	☐1 Always<br>☐2 Sometimes<br>☐3 Never	t807/t816
h. ATV/4-wheeler	☐1 Yes ☐3 No	☐1 Always<br>☐2 Sometimes<br>☐3 Never	t808/t817
i. Other (specify:______________________)<br>t809oth	☐1 Yes ☐3 No	☐1 Always<br>☐2 Sometimes<br>☐3 Never	t809/t818

2. Were the following pieces of equipment shared with another farm during the 14-day reference period? If Yes, how often were they cleaned and disinfected prior to returning to this farm?

**Equipment type**	**Shared with another farm in the 14-day reference period?**	**If Yes, how often was it cleaned and disinfected prior to returning to this farm?**	
a. Gates/panels	☐1 Yes ☐3 No	☐1 Always<br>☐2 Sometimes<br>☐3 Never	t819/t830
b. Lawn mowers	☐1 Yes ☐3 No	☐1 Always<br>☐2 Sometimes<br>☐3 Never	t820/t831
c. Live haul loaders	☐1 Yes ☐3 No	☐1 Always<br>☐2 Sometimes<br>☐3 Never	t821/t832
d. Catch pens	☐1 Yes ☐3 No	☐1 Always<br>☐2 Sometimes<br>☐3 Never	t822/t833
e. Scales for weighing birds	☐1 Yes ☐3 No	☐1 Always<br>☐2 Sometimes<br>☐3 Never	t823/t834
f. Vaccination equipment	☐1 Yes ☐3 No	☐1 Always<br>☐2 Sometimes<br>☐3 Never	t824/t835
g. Pressure sprayers/washers/foamers	☐1 Yes ☐3 No	☐1 Always<br>☐2 Sometimes<br>☐3 Never	t825/t836
h. Skid-steer loaders	☐1 Yes ☐3 No	☐1 Always<br>☐2 Sometimes<br>☐3 Never	t826/t837
i. Litter/manure handling	☐1 Yes ☐3 No	☐1 Always<br>☐2 Sometimes<br>☐3 Never	t827/t838
j. Tillers/de-caking equipment	☐1 Yes ☐3 No	☐1 Always<br>☐2 Sometimes<br>☐3 Never	t828/t839

k. Other (specify:____________________) t829oth	☐1 Yes ☐3 No	☐1 Always<br>☐2 Sometimes<br>☐3 Never	t829/t840

3. Considering the following equipment-related topics, how challenging would you say these are for producers to achieve? *[Check one box per row.]*

	Not at all challenging	Slightly challenging	Somewhat challenging	Quite challenging	Extremely challenging	
a. Keeping shared vehicles cleaned and disinfected	☐1	☐2	☐3	☐4	☐5	t841
b. Keeping shared small equipment (such as catch pens or litter tillers) cleaned and disinfected	☐1	☐2	☐3	☐4	☐5	t842
c. Other equipment or vehicle-related challenges (Specify: t843oth _______________)	☐1	☐2	☐3	☐4	☐5	t843

Section I – Litter Handling

1. Was fresh litter/bedding brought onto the farm during the 14-day reference period?

   t901 ☐1 Yes ☐3 No

   a. If Yes, who brought the fresh litter onto the farm? *[Check one only.]* t902

      ☐1 Company personnel

      ☐2 Litter provider

      ☐3 Other (specify: ____________________) t902oth

2. Is the fresh litter heat treated prior to delivery? t903 ☐1 Yes ☐3 No ☐4 Don't know

3. Prior to use, is fresh litter stored on the farm:

   a. Outside ....t904 ☐1 Yes ☐3 No

i. If Yes, is it covered? ....t905 ☐1 Yes ☐3 No

b. In a shed ....t906 ☐1 Yes ☐3 No

i. If Yes, is the shed closed? ....t907 ☐1 Yes ☐3 No

[If both Questions 3 a and b = No, SKIP to Question 6.]

4. What is the distance (in yards) from the on-site fresh litter storage area to the **selected barn**?
....t908 ________ yards

5. Prior to use, is fresh litter accessible to:

a. Wild birds ....t909 ☐1 Yes ☐3 No

b. Wild animals (for example, raccoons, opossum, coyotes, foxes)............t910 ☐1 Yes ☐3 No

c. Domestic animals (for example, dogs, cats) ....t911 ☐1 Yes ☐3 No

6. What was the date that used litter was last removed from any barn on this farm prior to the end of the 14-day reference period?.. ....t912 ________mm/dd/yy

7. How was used litter disposed of prior to or during the 14-day reference period?

a. Composted on-farm..t913 ☐1 Yes ☐3 No

i. If Yes, what is the distance (in yards) to the **selected bam**?............t914 _______ yards

b. Stored on-farm ....t915 ☐1 Yes ☐3 No

c. Applied to land on this farm ....t916 ☐1 Yes ☐3 No

i. If Yes, what was the date litter was applied to land? ....t917 _______ mm/dd/yy

d. Taken off-site ....t918 ☐1 Yes ☐3 No

8. Was manure or used litter from other farms brought onto this farm or adjacent farms prior to or during the reference period? t919 ☐1 Yes ☐3 No ☐4 Don't know

9. How many times was fresh litter added to the **selected barn** during the reference period?
t920 ________ times

These next three questions ask about the litter management practices for the selected barn around the time of the 14-day reference period.

10. Was litter "tilled" after it was placed in the **selected barn**?.............................t921 ☐1 Yes ☐3 No

11. Was there a partial clean out of the **selected barn**? ....t922 ☐1 Yes ☐3 No

12. When was the last full clean out of the **selected barn**? *[Check one only.]* t923

☐1 Prior to this flock

☐2 Two flocks ago

☐3 Three or more flocks ago

Section J – Dead Bird Disposal

1. What is the approximate normal daily mortality on this farm?.......................... t1001 _________ #/day

2. During the 14-day reference period, what were the method(s) of dead bird (daily mortality) disposal on this farm?

   a. Composting .... t1002 ☐1 Yes ☐3 No

   b. Burial.. t1003 ☐1 Yes ☐3 No

   c. Incineration .... t1004 ☐1 Yes ☐3 No

   d. Rendering .... t1005 ☐1 Yes ☐3 No

   e. Landfill .... t1006 ☐1 Yes ☐3 No

   f. Other (specify: __________________) t1007oth .... t1007 ☐1 Yes ☐3 No

3. If Question 2a (composting) or Question 2b (burial) is Yes, how frequently are carcasses covered with:

   a. Soil? .... t1008 ☐1 Daily ☐2 Every 2 or more days ☐3 Never

   b. Manure? .... t1009 ☐1 Daily ☐2 Every 2 or more days ☐3 Never

4. If Question 2d (rendering) is Yes,

   a. Is the carcass bin kept covered? .... t1010 ☐1 Yes ☐3 No

   b. Are carcasses: *[Check one only.]* t1011

      ☐1 Taken by the producer or worker to the renderer?

      ☐2 Picked up by the renderer from the farm?

   c. How many times were carcasses moved to the renderer during the 14-day reference period?

      t1012 _________ # times

5. Does this farm have an alternative mortality disposal plan if the typical method is disrupted and carcasses cannot be moved off farm?.. t1013 ☐1 Yes ☐3 No

6. Were any wild birds or wild mammals observed around the dead bird collection area (such as burial, compost pile, rendering bin, or similar) during the 14-day reference period?

   a. Wild birds .... t1014 ☐1 Yes ☐3 No

   b. Wild mammals.. t1015 ☐1 Yes ☐3 No

7. During the 14-day reference period, did this farm use a shared collection point for dead bird disposal? *[Check one only.]* t1016 ☐1 Yes – located on this farm ☐2 Yes – located off this farm ☐3 No

8. How far is the **selected barn** (in yards) from the dead bird disposal/holding area including carcass bin for rendering? .... t1017 _____ yards

Section K – Selected Barn Characteristics

Answer this entire section for the selected barn that was chosen in Section B. **Answer questions for the 14-day reference period.**

1. Which best describes the ground surface immediately surrounding (within 1 yard) this barn (excluding vehicle approach and loading area)? *[Check one only.]* t1101

   ☐1 Gravel or hard surface

   ☐2 Dirt

   ☐3 Short grass

   ☐4 Tall grass or brush

2. Were the following type(s) of poultry present in this barn during the 14-day reference period?

   a. Brooder .... t1102 ☐1 Yes ☐3 No

   b. Grower toms .... t1103 ☐1 Yes ☐3 No

   c. Grower hens .... t1104 ☐1 Yes ☐3 No

   d. Breeders .... t1105 ☐1 Yes ☐3 No

   e. Other (specify: ____________________________) t1106oth .... t1106 ☐1 Yes ☐3 No

3. For the flock that was present during the 14-day reference period, how many birds were placed in this barn? .... t1107____________ # birds

4. What was the date of placement in this barn? .... t1108__________mm/dd/yy

5. How old were birds when placed in this barn? t1109d ___________ days OR t1109w ______________weeks

6. Were different stages of production (for example, brooders and growers) present in this barn at the same time during the 14-day reference period? .... t1110 ☐1 Yes ☐3 No

7. Was there a partial load-out of this barn during the reference period? .... t1111 ☐1 Yes ☐3 No

8. Was there another health concern in this flock during the reference period? .... t1112 ☐1 Yes ☐3 No

   a. If Yes, specify condition: t1113 ____________________________________

9. Was this flock being treated for a condition or health concern during the reference period?

   t1114 ☐1 Yes ☐3 No

   a. If Yes, specify treatment: t1115____________________________________

10. How old is this barn structure? .... t1116 ________ years

11. How long has it been since the last remodel of the barn structure? .... t1117 ________ years

    t1117a ☐1 NA – Never remodeled

12. How well has the barn structure been maintained? *[Check one only.]* t1118

☐1 Well – For example, walls, curtains, and mud boards do not have holes, no visible daylight, the barn is tight and well insulated

☐2 Moderate – For example, barn could have rust or small holes, mud boards may be damaged, curtains may be torn or not in good repair, curtains may not close all the way, insulation may not be in good repair, the poly may be hanging from the ceiling

☐3 Poor – For example, holes in walls and mud boards are apparent, tin is rusted, may have leaks in roof, there might be some holes large enough for wild birds to enter, multiple areas with daylight visible, insulation may be hanging from the ceiling

13. Is this barn bird proof?.. t1119 ☐1 Yes ☐3 No

14. During the 14-day reference period, did you notice any water seepage into the barn (for example, water entering the barn from snowmelt or rainwater)? .... t1120 ☐1 Yes ☐3 No ☐4 Don't know

15. What type of ventilation was used for this barn during the 14-day reference period? *[Check one only.]* t1121

☐1 Curtain ventilated

☐2 Environmental control/tunnel ventilation

☐3 Side doors (such as tip outs)

☐4 Other (specify: ___________________) t1121oth

16. During the 14-day reference period,

a. What percentage of time were the curtains open? t1122/t1133 ________% time ☐4 Don't know

b. How many days were the curtains open or partially open? t1123/t1134 _____# days ☐4 Don't know

17. Was intake air filtered during the 14-day reference period? .... t1124 ☐1 Yes ☐3 No

a. If Yes, specify type of filter: t1125 ________________________________

18. During the 14-day reference period, was landscape fabric in place on either air intake inlets or along curtains on the barn?

a. On air intake inlets .... t1126 ☐1 Yes ☐3 No

b. Along curtains .... t1127 ☐1 Yes ☐3 No

[If both Question 18a and 18b = No, SKIP to Question 21.]

19. During the 14-day reference period, was any of this landscape fabric installed or replaced on either air intake inlets or along curtains? .... t1128 ☐1 Yes ☐3 No

20. During the 14-day reference period, was any of this landscape fabric sprayed with disinfectant on either air intake inlets or along curtains? .... t1129 ☐1 Yes ☐3 No

a. If Yes, how often was it sprayed: .... t1130/t1130a____times / ☐1 day OR ☐2 week

21. How frequently were the following used in this barn during the 14-day reference period? *[Check one per row.]*

	Used regularly	Not used regularly	Not available	
a. Cool cell pads	☐1	☐2	☐3	t1131
b. Misters	☐1	☐2	☐3	t1132

Section L – Office Use Only

1. Interview response code. *[Check only one.]* t1201

☐1 Survey completed

☐2 Refused

☐3 Out of business

☐4 No turkeys present during 2022

☐5 Inaccessible

☐6 Other (specify: ____________________) t1201oth

Comment Section

Please use this section for anything else that you would like to add. For example, how do you think HPAI was/is spreading within your geographic area? t1301
