# Supplementary material for: Investigation of risk factors for introduction of highly pathogenic avian influenza H5N1 infection among commercial turkey operations in the United States, 2022: a case-control study
Source: Front Vet Sci. 2023 Aug 30;10:1229071. doi: 10.3389/fvets.2023.1229071 (PMC10498466; doi:10.3389/fvets.2023.1229071)
Supplement: Supplementary file 2 [file Table_1.DOCX]

**Investigation of risk factors for wild bird introduction of highly pathogenic avian influenza H5N1 infection among commercial turkey operations in the United States, 2022: A case-control study**

**Supplementary Materials**

**Farm and Barn-Level Univariate Analysis**

**Table S1 - Premises description**

| **Characteristic** | **N (%) Case farms** | **N (%) Control farms** | **p-value**  (Fisher’s) |
| --- | --- | --- | --- |
| **In existing control zone*** | 38 (57.6) | 14 (23.7) | <0.001 |
| **Stage of production** |  |  |  |
| Brooder | 37 (56.1) | 24 (40.7) | 0.107 |
| Grower | 63 (95.5) | 51 (86.4) | 0.113 |
| **Sex** |  |  |  |
| Hens | 14 (21.2) | 28 (48.3) | 0.002 |
| Toms | 57 (86.4) | 39 (67.2) | 0.017 |
| **Age** |  |  |  |
| Multiple age | 34 (52.3) | 27 (47.4) | 0.717 |
| Single age | 31 (47.7) | 30 (52.6) |  |
| **Other type(s) of poultry on farm** | 13 (19.7) | 3 (5.2) | 0.017 |
| **Farm certified organic** | 7 (10.9) | 7 (12.5) | 1.000 |
| **Enrolled in NPIP** | 35 (63.6) | 37 (67.3) | 0.841 |
| If yes, enrolled in NPIP Avian Influenza Program | 29 (85.3) | 27 (87.1) | 1.000 |
| **Farm type** |  |  |  |
| Company farm | 14 (21.2) | 16 (27.6) | 0.119 |
| Independent farm | 29 (43.9) | 15 (25.9) |  |
| Contract farm/other | 23 (34.8) | 27 (46.6) |  |
| **Outdoors** |  |  |  |
| Any birds on the farm had access to outdoors* | ** | 3 (5.4) | 0.660 |
| Birds in selected barn had access to outdoors* | ** | 6 (10.3) | 0.050 |
| **Livestock, excluding poultry, on the farm or located within 320 m of farm fed supplemental feed*** | 11 (16.9) | 9 (15.5) | 1.000 |
| **Water source for poultry** |  |  |  |
| Off-site fresh water | 11 (16.7) | 10 (17.2) | 1.000 |
| Well | 53 (80.3) | 50 (86.2) | 0.474 |
| Surface water (e.g., pond) | ** | ** | 1.000 |
| **Water treatments used for drinking water** | 63 (95.5) | 54 (93.1) | 0.705 |
| If yes, given continuously | 60 (95.2) | 46 (86.8) | 0.182 |
| If yes, given intermittently | 3 (4.8) | 7 (13.2) |  |
| **Windbreak** |  |  |  |
| Any windbreak | 26 (40.0) | 16 (27.6) | 0.183 |
| Evergreen or juniper windbreak present | 12 (18.5) | 6 (10.3) | 0.307 |
| Deciduous tree windbreak present | 16 (25.0) | 10 (17.2) | 0.377 |
| Structural windbreak present | 9 (13.9) | 9 (15.5) | 0.804 |
| **Water body type(s) visible or within 320 m of farm** | 48 (72.7) | 36 (62.1) | 0.249 |
| Pond | 19 (28.8) | 11 (19.0) | 0.216 |
| Lake | 5 (7.6) | 3 (5.2) | 0.722 |
| Stream | 9 (13.6) | 6 (10.3) | 0.784 |
| River | 10 (15.2) | 5 (8.6) | 0.287 |
| Wetland or swamp | 17 (25.8) | 8 (13.8) | 0.119 |
| Wastewater lagoon | 5 (7.6) | 2 (3.4) | 0.447 |
| Standing water* | 6 (9.1) | 2 (3.4) | 0.281 |
| Drainage ditch or canal | 13 (19.7) | 15 (25.9) | 0.519 |
| **For waterbodies within 320 m of the farm, number of wild waterfowl or shorebirds seen on the water*** |  |  |  |
| None | 14 (29.2) | 17 (47.2) | 0.199 |
| Tens | 14 (29.2) | 7 (19.4) |  |
| Hundreds | 5 (10.4) | 2 (5.6) |  |
| Thousands | 4 (8.3) | 0 (0) |  |
| Don’t know | 11 (22.9) | 10 (27.8) |  |
| **Approximate number of wild waterfowl or shorebirds on closest body of water at one time*** |  |  |  |
| None | 18 (27.7) | 23 (41.1) | 0.102 |
| Tens | 20 (30.8) | 10 (17.9) |  |
| Hundreds | 6 (9.2) | 4 (7.1) |  |
| Thousands | 4 (6.2) | 0 (0) |  |
| Don’t know | 17 (26.2) | 19 (33.9) |  |
| **Crop last grown in closest field** |  |  |  |
| Corn | 24 (42.9) | 21 (52.5) | 0.574 |
| Soybeans | 21 (37.5) | 14 (35.0) |  |
| Alfalfa or grass intended for livestock feed | 11 (19.6) | 5 (12.5) |  |
| **Closest field tilled** |  |  |  |
| Fall 2021 | 27 (42.2) | 27 (46.6) | 0.536 |
| Spring 2022 | 23 (35.9) | 29 (50.9) | 0.090 |
| **Closest field actively worked (e.g., tilled)*** | 6 (9.8) | 6 (13.3) | 0.758 |
| **Approximate number of wild waterfowl or shorebirds seen in closest field*** |  |  |  |
| None | 32 (48.5) | 33 (56.9) | 0.060 |
| Tens | 10 (15.2) | 3 (5.2) |  |
| Hundreds | 5 (7.6) | 4 (6.9) |  |
| Thousands | 5 (7.6) | 0 (0) |  |
| Don’t know | 14 (21.2) | 18 (31.0) |  |

*Refers to the 14-day reference period.

**Too few to report.

**Table S2 - Wild birds***

| **Characteristic** | **Level** | **N (%) Case farms** | **N (%) Control farms** | **p-value**  (Fisher’s) |
| --- | --- | --- | --- | --- |
| **Frequency of wild birds seen on the farm and within 91.4 m of the outside of the barns** |  |  |  |  |
| Waterfowl (e.g., ducks, geese) | Often | 14 (21.9) | 2 (3.4) | 0.003 |
|  | Sometimes | 19 (29.7) | 14 (24.1) |  |
|  | Never | 31 (48.4) | 42 (72.4) |  |
| Gulls | Often | 9 (14.1) | 5 (8.6) | 0.700 |
|  | Sometimes | 10 (15.6) | 9 (15.5) |  |
|  | Never | 45 (70.3) | 44 (75.9) |  |
| Small perching birds (e.g., sparrows, starlings, swallows) | Often | 38 (60.3) | 31 (53.5) | 0.660 |
|  | Sometimes | 20 (31.8) | 20 (34.5) |  |
|  | Never | 5 (7.9) | 7 (12.1) |  |
| Blackbirds and crows | Often | 17 (27.0) | 19 (32.8) | 0.741 |
|  | Sometimes | 25 (39.7) | 23 (39.7) |  |
|  | Never | 21 (33.3) | 16 (27.6) |  |
| Other water birds (e.g., egrets, cormorants) | Often | 3 (4.7) | 0 (0) | 0.377 |
|  | Sometimes | 5 (7.8) | 4 (7.1) |  |
|  | Never | 56 (87.5) | 52 (92.9) |  |
| Wild turkeys, pheasants, quail | Often | 2 (3.1) | 3 (5.3) | 0.795 |
|  | Sometimes | 18 (28.1) | 14 (24.6) |  |
|  | Never | 44 (68.8) | 40 (70.2) |  |
| Raptors (e.g., eagles, hawks, owls, vultures) | Often | 8 (12.5) | 7 (12.1) | 0.935 |
|  | Sometimes | 32 (50.0) | 27 (46.6) |  |
|  | Never | 24 (37.5) | 24 (41.4) |  |
| Pigeons and doves | Often | 11 (17.5) | 10 (17.5) | 0.439 |
|  | Sometimes | 29 (46.0) | 20 (35.1) |  |
|  | Never | 23 (36.5) | 27 (47.4) |  |
| **Frequency of wild birds seen inside the selected barn** |  |  |  |  |
| Large birds (e.g., pigeons, crows) | Often/ sometimes | 3 (4.7) | 2 (3.4) | 1.000 |
|  | Never | 61 (95.3) | 56 (96.6) |  |
| Small birds (e.g., finches, sparrows, starlings) | Often | 2 (3.1) | 2 (3.4) | 0.852 |
|  | Sometimes | 11 (16.9) | 7 (12.1) |  |
|  | Never | 52 (80.0) | 49 (84.5) |  |
| **Sick or dead wild birds seen** |  |  |  |  |
| Large birds (e.g., pigeons, crows) | Inside the barns | 0 (0) | 0 (0) |  |
|  | Outside the barns | 3 (4.7) | 0 (0) | 0.246 |
| Small birds (e.g., finches, sparrows, starlings) | Inside the barns | 0 (0) | 0 (0) |  |
|  | Outside the barns | 4 (6.3) | 2 (3.6) | 0.684 |
| **If sick or dead wild birds were seen, they were:** |  |  |  |  |
| Left for predators |  | ** | ** | 0.464 |
| Disposed of by same method used for turkey mortality |  | ** | ** | 1.000 |
| Taken to rehab center, animal control or veterinarian |  | 0 (0) | 0 (0) |  |
| Other |  | 5 (62.5) | 0 (0) | 0.444 |

*All responses refer to the 14-day reference period.

**Too few to report.

**Table S3 - Farm biosecurity**

| **Characteristic** | **Level** | **N (%) Case farms** | **N (%) Control farms** | **p-value** (Fisher’s) |
| --- | --- | --- | --- | --- |
| **Road surface for vehicles coming onto operation** |  |  |  |  |
| Hard top/asphalt |  | 2 (3.1) | 2 (3.4) | 0.221 |
| Gravel |  | 59 (92.2) | 48 (82.8) |  |
| Dirt/Other |  | 3 (4.7) | 8 (13.8) |  |
| **Type of vehicle:** |  |  |  |  |
| Garbage/dumpster pick up | Come to perimeter | 24 (36.9) | 19 (32.2) | 0.935 |
|  | Enter farm but not near barns | 8 (12.3) | 9 (15.3) |  |
|  | Come near barns | 3 (4.6) | 3 (5.1) |  |
|  | Do not come | 30 (46.2) | 28 (47.5) |  |
| Propane delivery | Come to perimeter | 3 (4.6) | 0 (0) | 0.017 |
|  | Enter farm but not near barns | 21 (32.3) | 16 (27.6) |  |
|  | Come near barns | 13 (20.0) | 25 (43.1) |  |
|  | Do not come | 28 (43.1) | 17 (29.3) |  |
| Feed delivery | Come to perimeter | 4 (6.2) | 4 (6.8) | 0.100 |
|  | Enter farm but not near barns | 6 (9.2) | 0 (0) |  |
|  | Come near barns | 53 (81.5) | 53 (89.8) |  |
|  | Do not come | 2 (3.1) | 2 (3.4) |  |
| Feed ingredient delivery | Come to perimeter | 5 (7.9) | 2 (3.5) | 0.083 |
|  | Enter farm but not near barns or come near barns | 10 (15.9) | 3 (5.3) |  |
|  | Do not come | 48 (76.2) | 52 (91.2) |  |
| Renderer | Come to perimeter | 11 (17.7) | 7 (12.1) | 0.061 |
|  | Enter farm but not near barns | 3 (4.8) | 4 (6.9) |  |
|  | Come near barns | 6 (9.7) | 0 (0) |  |
|  | Do not come | 42 (67.7) | 47 (81.0) |  |
| Company personnel (e.g., catch crew, barn workers, service person, veterinarian) | Come to perimeter | 7 (10.6) | 6 (10.3) | 0.707 |
|  | Enter farm but not near barns | 8 (12.1) | 10 (17.2) |  |
|  | Come near barns | 40 (60.6) | 36 (62.1) |  |
|  | Do not come | 11 (16.7) | 6 (10.3) |  |
| Other business visitors (e.g., meter reader, repairman) | Come to perimeter | 8 (12.7) | 9 (15.8) | 0.581 |
|  | Enter farm but not near barns | 5 (7.9) | 7 (12.3) |  |
|  | Come near barns | 8 (12.7) | 10 (17.5) |  |
|  | Do not come | 42 (66.7) | 31 (54.4) |  |
| **Frequency vegetation mowed/bush hogged (times/month)** | Mean (standard deviation) | 3.1 (sd 1.4) | 3.3 (sd 1.8) | 0.563** |
|  | Less than 4 times a month | 31 (47.0) | 27 (45.8) | 1.000 |
|  | 4 or more times a month | 35 (53.0) | 32 (54.2) |  |
| **Wash station or spray area used for vehicles*** |  | 40 (60.6) | 40 (69.0) | 0.353 |
| If yes, was the vehicle wash station or spray area* | Located on the farm | 38 (97.4) | 36 (90.0) | 0.359 |
|  | Vehicle tires washed | 37 (92.5) | 39 (97.5) | 0.615 |
|  | Vehicle exterior washed | 14 (35.9) | 9 (22.5) | 0.222 |
|  | Vehicle interior cleaned | 20 (52.6) | 15 (38.5) | 0.256 |
|  | Worker vehicles washed | 31 (77.5) | 28 (70.0) | 0.460 |
|  | Feed trucks washed | 32 (80.0) | 35 (87.5) | 0.348 |
|  | Vehicles delivering or removing birds washed | 21 (52.5) | 22 (55.0) | 0.687 |
| Distance from vehicle wash station to selected barn (m)* | 68.6 m or less | 19 (51.4) | 16 (41.0) | 0.490 |
|  | Greater than 68.6 m | 18 (48.7) | 23 (59.0) |  |
|  |  |  |  |  |
| **Workers park in restricted area away from barns*** | Always | 41 (62.1) | 34 (59.6) | 0.949 |
|  | Sometimes | 3 (4.5) | 2 (3.5) |  |
|  | Never | 22 (33.3) | 21 (36.8) |  |
| **Visitors park in restricted area away from barns*** | Always | 39 (62.9) | 33 (56.9) | 0.291 |
|  | Sometimes | 2 (3.2) | 0 (0) |  |
|  | Never | 21 (33.9) | 25 (43.1) |  |
| **Animal/pest control** |  |  |  |  |
| Wild mammals or evidence seen in or around barns* |  | 13 (21.3) | 19 (35.2) | 0.144 |
| Rat and mouse bait stations used* |  | 66 (100.0) | 58 (100.0) |  |
| Beetle control used* |  | 43 (65.2) | 38 (66.7) | 1.000 |
| Fly control used* |  | 34 (52.3) | 26 (44.8) | 0.471 |
| Netting on barns to prevent wild bird access* |  | 49 (74.2) | 41 (71.9) | 0.840 |
| Wild birds able to access feed/feed ingredients* |  | 16 (24.2) | 15 (25.9) | 0.839 |
| Wild animals able to access feed/feed ingredients* |  | 8 (12.1) | 12 (20.7) | 0.227 |
| Rodents able to access feed/feed ingredients* |  | 17 (25.8) | 15 (25.9) | 1.000 |
| Written wildlife management plan in place |  | 41 (64.1) | 41 (74.6) | 0.239 |
| **Biosecurity audit/assessment conducted in previous 2 years** |  | 55 (84.6) | 44 (78.6) | 0.654 |

*Responses refer to the 14-day reference period.

**Score test statistic p-value.

**Table S4 - Selected barn biosecurity**

| **Characteristic** | **Level** | **N (%) Case farms** | **N (%) Control farms** | **p-value**  (Fisher’s) |
| --- | --- | --- | --- | --- |
| Frequency rodents observed* | Frequently (e.g., daily) | 3 (4.7) | 2 (3.5) | 0.715 |
|  | Occasionally (e.g., weekly) | 17 (26.6) | 19 (33.3) |  |
|  | Never | 44 (68.8) | 36 (63.2) |  |
| Intensity of beetles* | High | 3 (4.6) | 2 (3.4) | 0.796 |
|  | Medium | 2 (3.1) | 4 (6.9) |  |
|  | Low | 31 (47.7) | 26 (44.8) |  |
|  | None | 29 (44.6) | 26 (44.8) |  |
| Intensity of flies* | High | 7 (10.8) | 3 (5.2) | 0.659 |
|  | Medium | 8 (12.3) | 10 (17.2) |  |
|  | Low | 25 (38.5) | 22 (37.9) |  |
|  | None | 25 (38.5) | 23 (39.7) |  |
| Hard-surface entry pad |  | 58 (89.2) | 51 (87.9) | 1.000 |

* Responses refer to the 14-day reference period.

**Table S5 - Farm help/workers**

| **Characteristic** | **Level** | **N (%) Case farms** | **N (%) Control farms** | **p-value** (Fisher’s) |
| --- | --- | --- | --- | --- |
| **Use occasional or emergency workers to fill in during the 14-day reference period** |  | 10 (15.4) | 14 (24.6) | 0.256 |
| **Workers visit another poultry farm** |  | 9 (14.3) | 7 (12.1) | 0.896 |
| **Employed by other poultry operations, company farms, rendering plants, processing plants** | Workers | 0 (0) | 3 (5.3) | 0.243 |
|  | Members of household | 0 (0) | 5 (8.8) | 0.022 |
| **Employees own their own poultry, including backyard flocks** |  | * | * | 1.000 |
| **Employees required to stay off farm after exposure to other poultry** |  | 52 (83.9) | 43 (75.4) | 0.264 |
| **Time spent by all employees on farm biosecurity in a typical week** | 10 hours or less | 46 (76.7) | 29 (59.2) | 0.063 |
|  | More than 10 hours | 14 (23.3) | 20 (40.8) |  |

*Too few to report.

**Table S6 – Selected barn farm help/workers**

| **Characteristic** | **Level** | **N (%) Case farms** | **N (%) Control farms** | **p-value** (Fisher’s) | |
| --- | --- | --- | --- | --- | --- |
| **Measures used by workers entering the barn:*** |  |  |  |  | |
| Established clean/dirty line | Always | 55 (84.6) | 49 (86.0) | 0.864 | |
|  | Most of the time/ sometimes | 5 (7.7) | 3 (5.3) |  | |
|  | Never/not available | 5 (7.7) | 5 (8.8) |  | |
| Service room that personnel must enter through that separates “outside area” from “inside area” | Always/most of the time/sometimes | 56 (86.2) | 49 (86.0) | 0.454 | |
|  | Never | 5 (7.7) | 2 (3.5) |  | |
|  | NA – Not available | 4 (6.2) | 6 (10.5) |  | |
| Shower | Always | 3 (4.6) | 7 (12.1) | 0.049 | |
|  | Most of the time | 2 (3.1) | 0 (0) |  | |
|  | Sometimes | 2 (3.1) | 8 (13.8) |  | |
|  | Never | 28 (43.1) | 23 (39.7) |  | |
|  | NA – Not available | 30 (46.2) | 20 (34.5) |  | |
| Wash hands or use hand sanitizer before entering barn | Always | 37 (56.9) | 45 (77.6) | 0.153 | |
|  | Most of the time | 8 (12.3) | 4 (6.9) |  | |
|  | Sometimes | 9 (13.8) | 4 (6.9) |  | |
|  | Never | 9 (13.8) | 5 (8.6) |  | |
|  | NA – Not available | 2 (3.1) | 0 (0) |  | |
| Wear disposable gloves | Always | 33 (50.8) | 28 (48.3) | 0.992 | |
|  | Most of the time | 7 (10.8) | 6 (10.3) |  | |
|  | Sometimes | 11 (16.9) | 10 (17.2) |  | |
|  | Never | 11 (16.9) | 12 (20.7) |  | |
|  | NA – Not available | 3 (4.6) | 2 (3.4) |  | |
| Different personnel for different barns | Always | 16 (24.6) | 9 (15.5) | 0.682 | |
|  | Most of the time | 3 (4.6) | 4 (6.9) |  | |
|  | Sometimes | 10 (15.4) | 8 (13.8) |  | |
|  | Never | 23 (35.4) | 26 (44.8) |  | |
|  | NA – Not available | 13 (20.0) | 11 (19.0) |  | |
| Locks on barn doors | Always | 37 (57.8) | 28 (48.3) | 0.502 | |
|  | Most of the time | 2 (3.1) | 4 (6.9) |  | |
|  | Sometimes | 4 (6.3) | 2 (3.4) |  | |
|  | Never | 16 (25.0) | 21 (36.2) |  | |
|  | NA – Not available | 5 (7.8) | 3 (5.2) |  | |
| Wear disposable coveralls | Always | 24 (37.5) | 18 (31.0) | 0.752 | |
|  | Most of the time/ sometimes | 8 (12.5) | 11 (19.0) |  | |
|  | Never | 24 (37.5) | 21 (36.2) |  | |
|  | NA – Not available | 8 (12.5) | 8 (13.8) |  | |
| Change of clothing/coveralls (washable) | Always | 44 (67.7) | 36 (62.1) | 0.655 | |
|  | Most of the time | 3 (4.6) | 2 (3.4) |  | |
|  | Sometimes | 4 (6.2) | 8 (13.8) |  | |
|  | Never | 12 (18.5) | 9 (15.5) |  | |
|  | NA – Not available | 2 (3.1) | 3 (5.2) |  | |
| Change of shoes or use of shoe covers | Always | 59 (90.8) | 54 (93.1) | 0.748 | |
|  | Not always | 6 (9.2) | 4 (6.9) |  | |
| Scrub footwear (bucket and brush) | Always | 27 (41.5) | 23 (39.7) | 0.771 | |
|  | Most of the time | 0 (0) | 0 (0) |  | |
|  | Sometimes | 9 (13.8) | 8 (13.8) |  | |
|  | Never | 23 (35.4) | 18 (31.0) |  | |
|  | NA – Not available | 6 (9.2) | 9 (15.5) |  | |
| Foot bath (liquid) | Always | 48 (75.0) | 42 (75.0) | 0.883 | |
|  | Most of the time | 2 (3.1) | 3 (5.4) |  | |
|  | Sometimes | 0 (0) | 0 (0) |  | |
|  | Never/not available | 14 (21.9) | 11 (19.6) |  | |
| Foot bath (dry, such as powdered or particulate) | Always | 21 (33.3) | 16 (30.8) | 0.947 | |
|  | Most of the time/ sometimes | 5 (7.9) | 4 (7.7) |  | |
|  | Never | 20 (31.7) | 15 (28.8) |  | |
|  | NA – Not available | 17 (27.0) | 17 (32.7) |  | |
| **Frequency liquid or dry footbaths changed/Month*** | Mean (standard deviation) | 21.6  (sd 18.7) | 22.7  (sd 24.8) | | 0.803** |

*Responses refer to the 14-day reference period.

**Score test statistic p-value.

**Table S7 – Farm and selected barn visitors during the 14-day reference period**

| **Visitor type** | **Visited farm/ entered selected barn* during the 14-day reference period** | **N (%) Case farms** | **N (%) Control farms** | **p-value**  (Fisher’s) |
| --- | --- | --- | --- | --- |
| Federal/State veterinary or animal health worker | Visited farm | 4 (6.3) | 4 (6.9) | 1.000 |
|  | Entered selected barn | 2 (50.0) | 3 (75.0) | 1.000 |
| Extension agent or university veterinarian | Visited farm | 0 (0) | 0 (0) |  |
|  | Entered selected barn | - | - |  |
| Private or company veterinarian | Visited farm | 8 (12.5) | 7 (12.3) | 1.000 |
|  | Entered selected barn | 4 (57.1) | 5 (83.3) | 0.559 |
| Company service person | Visited farm | 22 (33.8) | 36 (62.1) | 0.002 |
|  | Entered selected barn | 20 (90.9) | 30 (96.8) | 0.563 |
| Nutritionist or feed company consultant | Visited farm | 6 (9.5) | ** | 0.117 |
|  | Entered selected barn | 2 (33.3) | ** | 0.429 |
| Bird delivery personnel (e.g., poult placement, move brood to grow) | Visited farm | 10 (15.9) | 10 (17.9) | 0.810 |
|  | Entered selected barn | 0 (0) | 3 (33.3) | 0.206 |
| Vaccination crew | Visited farm | 0 (0) | 0 (0) |  |
|  | Entered selected barn | - | - |  |
| Catch crew (bird removal) | Visited farm | ** | 4 (7.0) | 0.422 |
|  | Entered selected barn | ** | 3 (75.0) | 1.000 |
| Feed ingredient delivery person | Visited farm | 3 (4.8) | 7 (13.0) | 0.184 |
|  | Entered selected barn | 0 (0) | ** | 1.000 |
| Feed delivery personnel | Visited farm | 47 (73.4) | 49 (84.5) | 0.184 |
|  | Entered selected barn | 2 (4.4) | 11 (23.4) | 0.014 |
| Fresh litter delivery services | Visited farm | 4 (6.3) | 3 (5.4) | 1.000 |
|  | Entered selected barn | 0 (0) | 0 (0) |  |
| Litter removal services (e.g., litter broker, litter disposal) | Visited farm | ** | ** | 1.000 |
|  | Entered selected barn | ** | ** |  |
| Customer (private individual) | Visited farm | 0 (0) | ** | 0.475 |
|  | Entered selected barn | - | ** |  |
| Wholesaler, buyer, or dealer | Visited farm | 0 (0) | ** | 0.471 |
|  | Entered selected barn | - | 0 (0) |  |
| Renderer | Visited farm | 7 (11.1) | 5 (8.8) | 0.766 |
|  | Entered selected barn | ** | ** | 1.000 |
| Dead bird pickup other than by renderer | Visited farm | 10 (15.6) | 5 (8.8) | 0.283 |
|  | Entered selected barn | 2 (28.6) | 0 (0) | 0.462 |
| Rodent control crew | Visited farm | 3 (4.8) | ** | 0.621 |
|  | Entered selected barn | ** | ** | 1.000 |
| Occasional worker (e.g., family member, part-time help over holiday) | Visited farm | 9 (14.1) | 9 (15.5) | 1.000 |
|  | Entered selected barn | 7 (87.5) | 9 (100.0) | 0.471 |
| Construction workers, repair or maintenance personnel | Visited farm | 3 (4.7) | ** | 0.621 |
|  | Entered selected barn | 0 (0) | ** | 0.250 |
| Other business visitors (e.g., other producers, meter readers) | Visited farm | 5 (8.1) | 6 (10.3) | 0.757 |
|  | Entered selected barn | 0 (0) | 0 (0) |  |
| Other nonbusiness visitors (including neighbors, family members, friends, school field trips) | Visited farm | 0 (0) | 0 (0) |  |
|  | Entered selected barn | - | - |  |

*Among farms that had the visitor, percentage where the visitor entered the selected barn.

**Too few to report.

**Table S8 - Farm and selected barn visitors**

| **Characteristic** | **Level** | **N (%) Case farms** | **N (%) Control farms** | **p-value** (Fisher’s) |
| --- | --- | --- | --- | --- |
| **Frequency visitor log used** | Always | 39 (60.0) | 45 (77.6) | 0.005 |
|  | Sometimes | 15 (23.1) | 2 (3.4) |  |
|  | Never | 11 (16.9) | 11 (19.0) |  |
| **Requirements of visitors who entered the selected barn during the 14-day reference period:** |  |  |  |  |
| Change of outer clothing/farm specific clothing/coveralls | Required, verified at farm | 27 (58.7) | 27 (54.0) | 0.545 |
|  | Required, visitor responsibility | 6 (13.0) | 11 (22.0) |  |
|  | Not required | 13 (28.3) | 12 (24.0) |  |
| Foot covers or change of footwear | Required, verified at farm | 28 (60.9) | 31 (62.0) | 1.000 |
|  | Required, visitor responsibility | 11 (23.9) | 12 (24.0) |  |
|  | Not required | 7 (15.2) | 7 (14.0) |  |
| Mask | Required, verified at farm | 21 (45.7) | 15 (30.0) | 0.176 |
|  | Required, visitor responsibility | 6 (13.0) | 13 (26.0) |  |
|  | Not required | 19 (41.3) | 22 (44.0) |  |
| Hand sanitizing or handwashing | Required, verified at farm | 21 (45.7) | 27 (54.0) | 0.694 |
|  | Required, visitor responsibility | 11 (23.9) | 11 (22.0) |  |
|  | Not required | 14 (30.4) | 12 (24.0) |  |
| Gloves | Required, verified at farm | 23 (50.0) | 24 (48.0) | 0.713 |
|  | Required, visitor responsibility | 7 (15.2) | 11 (22.0) |  |
|  | Not required | 16 (34.8) | 15 (30.0) |  |
| Not visit multiple farms in a day | Required, verified at farm | 19 (42.2) | 20 (40.8) | 0.322 |
|  | Required, visitor responsibility | 9 (20.0) | 16 (32.7) |  |
|  | Not required | 17 (37.8) | 13 (26.5) |  |
| **Restroom facility (including portable) available to crews visiting farm** | Always (24 hours/day) | 27 (48.2) | 32 (60.4) | 0.014 |
|  | Sometimes | 3 (5.4) | 9 (17.0) |  |
|  | Never | 26 (46.4) | 12 (22.6) |  |

**Table S9 - Farm vehicles and equipment**

| **Factor** | **Level** | **N (%) Case farms** | **N (%) Control farms** | **p-value** (Fisher’s) |
| --- | --- | --- | --- | --- |
| **Vehicles shared during the 14-day reference period:** |  |  |  |  |
| Company trucks or trailers (e.g., pickup truck, trailer, supervisor truck) | Shared, always disinfected | 7 (11.3) | 9 (16.4) | 0.589 |
|  | Shared, sometimes/never disinfected | 2 (3.2) | 3 (5.5) |  |
|  | Not shared | 53 (85.5) | 43 (78.2) |  |
| Feed trucks | Shared, always disinfected | 22 (40.7) | 19 (40.4) | 0.067 |
|  | Shared, sometimes disinfected | 5 (9.3) | 0 (0) |  |
|  | Shared, never disinfected | 2 (3.7) | 0 (0) |  |
|  | Not shared | 25 (46.3) | 28 (59.6) |  |
| Feed ingredient truck | Not shared | 58 (92.1) | 55 (98.2) | 0.212 |
| Bird delivery vehicles (e.g., placing birds) | Shared, always disinfected | 4 (6.7) | 5 (8.6) | 0.612 |
|  | Shared, sometimes disinfected | 0 (0) | * |  |
|  | Shared, never disinfected | 0 (0) | * |  |
|  | Not shared | 56 (93.3) | 52 (89.7) |  |
| Bird removal vehicles (e.g., moved to slaughter, moved to grow) | Shared, always disinfected | 4 (6.3) | 4 (6.9) | 1.000 |
|  | Shared, sometimes disinfected | 0 (0) | 0 (0) |  |
|  | Shared, never disinfected | 0 (0) | 0 (0) |  |
|  | Not shared | 59 (93.7) | 54 (93.1) |  |
| Manure/litter hauling | Not shared | 63 (98.4) | 55 (94.8) | 0.346 |
| ATV/4-wheeler | Not shared | 64 (100.0) | 56 (96.6) | 0.224 |
| **Equipment shared during the 14-day reference period:** |  |  |  |  |
| Gates/panels | Not shared | 62 (100.0) | 55 (98.2) | 0.475 |
| Lawn mowers | Not shared | 60 (96.8) | 55 (98.2) | 1.000 |
| Live haul loaders | Shared, always disinfected | 3 (5.0) | 3 (5.4) | 1.000 |
|  | Shared, sometimes disinfected | 0 (0) | 0 (0) |  |
|  | Shared, never disinfected | 0 (0) | 0 (0) |  |
|  | Not shared | 57 (95.0) | 53 (94.6) |  |
| Catch pens | Not shared | 62 (100.0) | 56 (100.0) |  |
| Scales for weighing birds | Not shared | 62 (100.0) | 55 (98.2) | 0.475 |
| Pressure sprayers/ washers/foamers | Shared, always disinfected | * | 3 (5.4) | 0.345 |
|  | Shared, sometimes disinfected | * | 0 (0) |  |
|  | Shared, never disinfected | 0 (0) | 0 (0) |  |
|  | Not shared | 61 (98.4) | 53 (94.6) |  |
| Skid-steer loaders | Not shared | 59 (95.2) | 54 (96.4) | 1.000 |
| Litter/manure handling | Shared, always disinfected | * | 4 (7.3) | 0.186 |
|  | Shared, sometimes disinfected | * | 0 (0) |  |
|  | Shared, never disinfected | 0 (0) | 0 (0) |  |
|  | Not shared | 61 (98.4) | 51 (92.7) |  |
| Tillers/de-caking equipment | Not shared | 61 (98.4) | 55 (100.0) | 1.000 |

*Too few to report.

**Table S10 - Farm litter handling**

| **Characteristic** | **Level** | **N (%) Case farms** | **N (%) Control farms** | **p-value**  (Fisher’s) |
| --- | --- | --- | --- | --- |
| Fresh litter/bedding brought onto farm* |  | 11 (16.9) | 9 (15.5) | 1.000 |
| If yes, person who brought fresh litter on | Company personnel | 5 (55.6) | 3 (42.9) | 1.000 |
|  | Litter provider | 4 (44.4) | 4 (57.1) |  |
| Litter heat treated prior to delivery |  | 15 (23.1) | 16 (27.6) | 0.713 |
| Litter stored on farm | Stored outside | 2 (3.3) | 3 (5.2) | 0.674 |
|  | Not stored outside | 59 (96.7) | 55 (94.8) |  |
|  | Stored in a shed | 19 (30.6) | 16 (28.1) | 0.841 |
|  | Not stored in a shed | 43 (69.4) | 41 (71.9) |  |
| Distance on-site fresh litter storage to selected barn | Less than 91.4 m | 6 (31.6) | 8 (47.1) | 0.496 |
|  | 91.4 m or more | 13 (68.4) | 9 (52.9) |  |
| Prior to use, fresh litter accessible to: | Wild birds | 3 (16.7) | 6 (33.3) | 0.443 |
|  | Wild animals | 2 (11.1) | 6 (33.3) | 0.229 |
|  | Domestic animals | 2 (11.1) | 4 (22.2) | 0.658 |
| Used litter disposal | Composted on-farm | 12 (18.8) | 9 (16.1) | 0.811 |
|  | Stored on-farm | 12 (18.8) | 11 (19.6) | 1.000 |
|  | Applied to land on farm | 18 (28.1) | 10 (17.9) | 0.202 |
|  | Taken off-site | 44 (68.8) | 40 (71.4) | 0.843 |
| Manure/used litter brought onto this farm or adjacent farms* |  | ** | ** | 1.000 |

*Responses refer to the 14-day reference period.

**Too few to report.

**Table S11 - Litter management for selected barn around the time of the 14-day reference period**

| **Practice** | **Level** | **N (%) Case farms** | **N (%) Control farms** | **p-value**  (Fisher’s) |
| --- | --- | --- | --- | --- |
| Litter tilled after placed in barn |  | 15 (23.1) | 24 (41.4) | 0.034 |
| Partial clean out |  | 5 (7.8) | 6 (10.3) | 0.755 |
| Last full clean out | Prior to this flock | 44 (71.0) | 36 (65.5) | 0.163 |
|  | Two flocks ago | 10 (16.1) | 5 (9.1) |  |
|  | Three or more flocks ago | 8 (12.9) | 14 (25.5) |  |

**Table S12 - Dead bird disposal**

| **Characteristic** | **Level** | **N (%) Case farms** | **N (%) Control farms** | **p-value**  (Fisher’s) |
| --- | --- | --- | --- | --- |
| **Methods(s) of daily mortality disposal:*** |  |  |  |  |
| Composting |  | 33 (51.6) | 26 (44.8) | 0.474 |
|  | Covered daily or every 2 days with soil | 5 (18.5) | 11 (47.8) | 0.036 |
|  | Never covered with soil | 22 (81.5) | 12 (52.2) |  |
|  | Covered daily with manure | 24 (80.0) | 15 (71.4) | 0.507 |
|  | Covered every 2 or more days with manure | 4 (13.3) | 2 (9.5) |  |
|  | Never covered with manure | 2 (6.7) | 4 (19.0) |  |
| Burial |  | 8 (12.5) | 10 (17.2) | 0.610 |
|  | Covered daily with soil | 6 (75.0) | 8 (80.0) | 0.765 |
|  | Covered every 2 or more days with soil | ** | 0 (0) |  |
|  | Never covered with soil | ** | 2 (20.0) |  |
|  | Covered daily with manure | ** | ** | 0.478 |
|  | Covered every 2 or more days with manure | ** | ** |  |
|  | Never covered with manure | 3 (60.0) | 7 (87.5) |  |
| Incineration |  | 4 (6.3) | 10 (17.2) | 0.086 |
| Rendering |  | 18 (28.1) | 8 (13.8) | 0.076 |
|  | Carcass bin kept covered | 16 (88.9) | 8 (100.0) | 1.000 |
|  | Producer/worker takes carcasses to renderer | 4 (25.0) | ** | 0.631 |
|  | Carcasses picked up from farm by renderer | 12 (75.0) | 7 (87.5) |  |
| Landfill |  | ** | 4 (6.9) | 0.190 |
| **Alternative mortality disposal plan in place for farm** |  | 36 (58.1) | 27 (47.4) | 0.273 |
| **Wild birds seen around dead bird collection area*** |  | 20 (32.8) | 18 (31.6) | 1.000 |
| **Wild mammals seen around dead bird collection area*** |  | 9 (15.0) | 9 (15.8) | 1.000 |
| **Shared collection point for dead bird disposal*** | Yes, located on farm | 5 (7.9) | 4 (7.0) | 0.370 |
|  | Yes, located off farm | 5 (7.9) | ** |  |
|  | No | 53 (84.1) | 52 (91.2) |  |
| **Distance from selected barn to dead bird disposal/holding area** | Less than 137.2 m | 27 (49.1) | 25 (49.0) | 1.000 |
|  | 137.2 m or more | 28 (50.9) | 26 (51.0) |  |

*Responses refer to practices during the 14-day reference period.

**Too few to report.

**Table S13 - Selected barn characteristics during the 14-day reference period**

| **Characteristic** | | **Level** | **N (%) Case farms** | **N (%) Control farms** | **p-value**  (Fisher’s) |
| --- | --- | --- | --- | --- | --- |
| Ground surface immediately surrounding barn | | Gravel or hard surface | 27 (42.2) | 25 (43.1) | 1.000 |
|  |  | Dirt | 11 (17.2) | 10 (17.2) |  |
|  |  | Short grass/tall grass or brush | 26 (40.6) | 23 (39.7) |  |
| Poultry types present in barn | | Brooder | 11 (17.2) | 15 (26.3) | 0.270 |
|  |  | Grower toms | 51 (79.7) | 27 (47.4) | <0.001 |
|  |  | Grower hens | 7 (10.9) | 18 (31.6) | 0.007 |
| Different stages of production present in barn at same time | |  | * | 5 (8.9) | 0.098 |
| Partial load-out | |  | 3 (4.8) | * | 0.621 |
| Another health concern in the flock | |  | 6 (9.5) | 4 (7.0) | 0.746 |
| Flock being treated for condition or health concern | |  | 4 (6.3) | 3 (5.3) | 1.000 |
| Maintenance of barn structure | | Well | 51 (79.7) | 48 (82.8) | 0.817 |
|  |  | Moderate/poor | 13 (20.3) | 10 (17.2) |  |
| Barn is bird proof | |  | 57 (89.1) | 53 (91.4) | 0.766 |
| Water seepage into the barn during the 14-day reference period | |  | 10 (15.6) | 5 (8.6) | 0.350 |
| Ventilation type | | Curtain | 29 (46.0) | 22 (40.7) | 0.904 |
|  |  | Environmental/tunnel | 16 (25.4) | 14 (25.9) |  |
|  |  | Side doors (such as tip outs) | 3 (4.8) | 4 (7.4) |  |
|  |  | Other | 15 (23.8) | 14 (25.9) |  |
| Air intake filtered | |  | 11 (17.7) | 11 (21.2) | 0.812 |
| Landscape fabric in place | | Yes, on air intake inlets | 8 (13.1) | 7 (12.7) | 1.000 |
|  |  | Yes, along curtains | 13 (21.0) | 16 (29.1) | 0.392 |
| If yes, landscape fabric installed or replaced on either air intake inlets or along curtains |  | 2 (11.1) | 0 (0) | 0.486 |  |
| If yes, landscape fabric sprayed with disinfectant on either air intake inlets or along curtains | |  | 10 (55.6) | 8 (44.4) | 0.740 |
| Frequency of cool cell pad use | | Used regularly/not used regularly | 11 (18.3) | 9 (16.1) | 0.809 |
|  |  | Not available | 49 (81.7) | 47 (83.9) |  |
| Frequency of mister use | | Used regularly | 5 (8.1) | 7 (12.5) | 0.664 |
|  |  | Not used regularly | 25 (40.3) | 19 (33.9) |  |
|  |  | Not available | 32 (51.6) | 30 (53.6) |  |

*Too few to report.
